# Equivalence analysis to support environmental safety assessment: Using nontarget organism count data from field trials with cisgenically modified potato

**DOI:** 10.1002/ece3.4964

**Published:** 2019-02-14

**Authors:** Hilko van der Voet, Paul W. Goedhart, Jenny Lazebnik, Geert J. T. Kessel, Ewen Mullins, Joop J. A. van Loon, Salvatore Arpaia

**Affiliations:** ^1^ Wageningen University & Research Wageningen The Netherlands; ^2^ Crop Science Department Teagasc Carlow Ireland; ^3^ Energy and Environment Research Center ENEA, Italian National Agency for New Technologies Trisaia di Rotondella Italy

**Keywords:** environmental risk assessment, equivalence test, hierarchical analysis, limit of concern, multicriteria decision analysis, overdispersed Poisson

## Abstract

This paper considers the statistical analysis of entomological count data from field experiments with genetically modified (GM) plants. Such trials are carried out to assess environmental safety. Potential effects on nontarget organisms (NTOs), as indicators of biodiversity, are investigated. The European Food Safety Authority (EFSA) gives broad guidance on the environmental risk assessment (ERA) of GM plants. Field experiments must contain suitable comparator crops as a benchmark for the assessment of designated endpoints. In this paper, a detailed protocol is proposed to perform data analysis for the purpose of assessing environmental safety. The protocol includes the specification of a list of endpoints and their hierarchical relations, the specification of intended levels of data analysis, and the specification of provisional limits of concern to decide on the need for further investigation. The protocol emphasizes a graphical representation of estimates and confidence intervals for the ratio of mean abundances for the GM plant and its comparator crop. Interpretation relies mainly on equivalence testing in which confidence intervals are compared with the limits of concern. The proposed methodology is illustrated with entomological count data resulting from multiyear, multilocation field trials. A cisgenically modified potato line (with enhanced resistance to late blight disease) was compared to the original conventional potato variety in the Netherlands and Ireland in two successive years (2013, 2014). It is shown that the protocol encompasses alternative schemes for safety assessment resulting from different research questions and/or expert choices. Graphical displays of equivalence testing at several hierarchical levels and their interpretation are presented for one of these schemes. The proposed approaches should be of help in the ERA of GM or other novel plants.

## 1 INTRODUCTION

Genetically modified (GM) crops must undergo an environmental risk assessment (ERA) as part of a procedure to decide whether they can be admitted to the European market. Perry et al. (2009) and the EFSA Guidance on the ERA of GM plants (EFSA, 2010b) give broad guidance on the design of field experiments and subsequent data analysis to assess potential environmental impacts. In field studies, one of the main questions is whether the GM organism (GMO) is substantially equivalent to a comparator (CMP) when considering biodiversity as represented by assemblages of nontarget organisms (NTOs) linked to the receiving agro‐ecosystem. This comparative assessment is usually based on a large number of taxa (individual species or guilds, Arpaia, 2010). The outcome of the risk assessment is therefore not straightforward, and attempts to summarize results may lead to misleading conclusions (Devos, Schrijver, Clercq, Kiss, & Romeis, 2012).

The ERA Guidance document (EFSA, 2010b) identified the impact on NTOs as one of the areas of concern and requires the application of appropriate statistical procedures. However, the document does not give specific examples and solutions for practical problems in real case studies. For example, the Guidance states that “it is essential to specify for each variable studied a minimum effect size which is considered to potentially have a relevant impact on the receiving environment(s),” but does not indicate how to do this for low‐abundance species with highly variable counts. There is also no guidance whether counts should be added over different time points in a season or analyzed separately. As another example, the Guidance states that the “main analysis shall address all field trials simultaneously and shall be based on the full dataset from all sites.” However, this disregards the common problem that experimental procedures and even identified species are likely to be different for different experiments. The EU‐funded research project AMIGA—Assessing and Monitoring Impacts of Genetically modified plants on Agro‐ecosystems (Arpaia et al., 2014)—has performed research to apply the general EFSA Guidance to specific examples involving maize and potato field trials. Detailed proposals on how to conduct an ERA following the general guidance in specific cases are reported in this paper.

The practical possibilities for conducting ERA‐related field trials are diverse across the multiple biogeographical regions or receiving environments in Europe (Arpaia et al., 2014). As a consequence, the identified taxa are typically different between field trials, for example, because some taxa are restricted to certain regions, or because identification of arthropods depends on the specialized expertise that is available locally. It may therefore be preferable to analyze NTO abundances in terms of functional categories or guilds, but without losing attention for important individual indicator taxa at specific locations. A hierarchical analysis to deal with such issues is therefore needed. A proposal for a framework for hierarchical assessment is given in this paper.

A statistical analysis of comparative field trials comes in two flavors: difference testing and equivalence testing. Most research intends to find differences between treatments or groups, and the null hypothesis of the usual statistical tests states that group means are equal. Rejecting this null hypothesis is then considered a proof for the existence of differences. In contrast, safety assessments have the intention to show the absence of relevant effects. Therefore, we propose to employ equivalence testing which aims at rejecting the null hypothesis that the difference between the GM plant and its comparator exceeds a limit of concern (LOC). Rejection of this nonequivalence hypothesis implies that the difference is smaller than the LOC, and this can be considered as a proof of safety (Bross, 1985; Hothorn & Oberdoerfer, 2006; Millard, 1987; Perry et al., 2009). The advantages of using the equivalence concept for safety assessment have been described before (e.g., Perry et al., 2009; van der Voet, Perry, Amzal, & Paoletti, 2011; Meyners, 2012; Kang & Vahl, 2014; Goedhart, Voet, Baldacchino, & Arpaia, 2014; Vahl & Kang, 2016). A crucial argument in favor of equivalence testing is that the onus to do high‐quality, well‐replicated experiments with sufficient statistical power is placed on to those who wish to demonstrate the safety of GMOs (Perry et al., 2009). A flexible system to set limits of concern is proposed in this paper. It addresses a commonly encountered problem in entomological surveys, which is the occurrence of taxa with many zero catches (per plot) and perhaps only a few specimens overall. We also advise on the statistical model to analyze count data.

In the remainder of this introductory section, we address in more detail two general issues: Section 1.1 deals with the explicit research questions in relation to the hierarchical nature of the entomological data and Section 1.2 with the need to specify limits of concern for the chosen endpoints. In Section 2, we present the theory and motivation for a proposal for a statistical analysis methodology. This proposal is illustrated with a practical example in Section 3. Finally, in Section 4 the proposed methodology is discussed in the context of ERA.

### 1.1 Research questions and a hierarchy of endpoints

**Figure 1:**
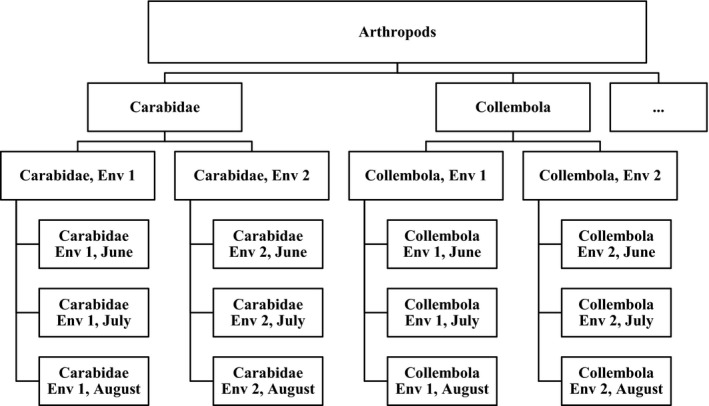
Simplified example of a hierarchy of endpoints in which the two endpoints Carabidae and Collembola are sampled in different environments at different points in time during the season. Env: Environment

When designing an experiment, it is essential to have a clear description of the research questions at hand and the proposed methodology to answer these questions. For an operational procedure concerning NTOs in a GM crop field trial, it is necessary to specify a list of endpoints that will be measured. Here, “endpoint” can be understood at several levels. For example, the endpoint “Carabidae” may refer to the total of pitfall trap catches of carabid beetles per plot over the field season in an intended single‐environment experiment, but it may also refer to the catch per plot at one specific sampling time in spring (a more refined level) or the average catch per plot over multiple environments (a more integrated level). In general, it will be possible to arrange these levels hierarchically, as shown for a simplified example in Figure 1.

“Environment” here can denote another site or another year, or both. The logical tree also shows further integration of endpoints into a larger “Arthropods” category. Risk assessors should establish at which level they pose their research question. For example,

- Is there a potential concern if the GM crop would affect the carabids in August in one specific environment, or
- Is it sufficient to consider the total count of carabids over a year for this environment, or
- Is it sufficient to consider the average carabid counts over multiple environments, or
- Can the research question be framed in terms of counts for functional groups, like predators and herbivores, or even all arthropods?

In the data analysis, we can distinguish three parts:

1. preprocessing of the data, for example, logarithmic transformations, but also integration steps such as summing pitfall trap catches over all time points in the field season;
2. the intended method of statistical analysis (SA) to estimate effects, that is, the differences between crop genotypes, from the data, as will be further discussed in Section 2 and Section 2.5;
3. the intended method of equivalence analysis (EA) to integrate estimated effects or concerns to higher levels in the hierarchy.

**Figure 2:**
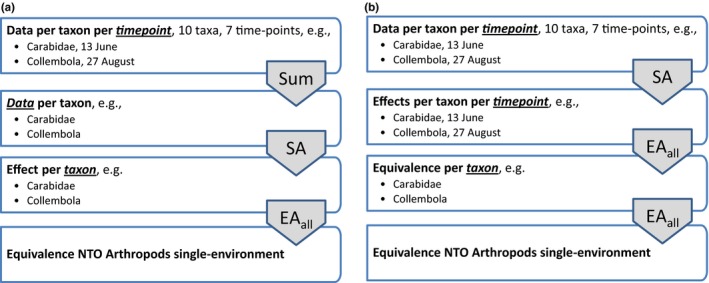
Two simple examples (a and b) of alternative logical trees for analysis of arthropod count data in a single‐environment NTO study of a GM crop compared to a comparator variety. EA_all_: equivalence analysis requiring all concern quotients to be within limits; SA: statistical analysis; Sum: summation of data

Figure 2 gives two examples of the structure of an intended data analysis for a single field experiment designed to compare a new genotype to a comparator variety or genotype. Suppose that there are counts for ten arthropod taxa and that data will be collected at seven time points during the field season. A possible choice, as in hierarchy A, may be not to study the endpoints at the time points level, but only at the level of the season total counts. This is especially practical for rare taxa. Summing is indicated by the “Sum” preprocessing step; the underlining of “time point” is meant to indicate that in this step some kind of summary over timepoints is made. After this, the data will be analyzed in a statistical analysis (SA step) to provide estimates and confidence intervals for the ten effects (differences between the tested genotype and its comparator). The statistical analysis would normally involve an ANOVA type of analysis. Underlining of “Data” indicates that Data are summarized to give estimated effects. After this step, each of the effects can be judged for equivalence. In the final step, denoted by EA_all_, the equivalence for the individual taxa is combined in an overall NTO equivalence assessment. The subscript “all” in EA_all_ denotes that each individual endpoint should meet its equivalence criterion and “taxon” is therefore underlined.

Hierarchy B in Figure 2 presents an alternative. Here, the data are analyzed at the time points level, and application of EA_all_ in two steps now requires that the observed effects at all time points should fulfill the equivalence criteria. Note that the counts in the statistical analysis in B will be much lower as compared to hierarchy A, and therefore, it will be more difficult to have sufficient power for all 10 × 7 = 70 endpoints. In fact, scheme B may not be practical at all, when it is expected that some species are not present (expected counts zero) during parts of the field season. In principle, the scheme could be adapted by specifying for each taxon the relevant time intervals during the season. The first EA_all_ step in hierarchy B could be replaced by a less strict requirement that each taxon should only on average meet the equivalence limits during the growing season. An EA_av _(av for average) equivalence analysis will be more precisely defined in Section 2.4.

The key message of this simple example is that alternative logical hierarchies for the analysis are possible and that these choices can have a big impact on the number of required replications and thus on the cost‐benefit reasoning relevant for the planning of field studies. Hierarchy B, for example, will require more replications than hierarchy A because it is required that equivalence is met for every time‐point rather than for the sum across time points. Further details of the data analysis methods are discussed in relation to the proposed statistical analysis protocol (Sections 2.1 and 2.5).

### 1.2 Limits of concern

The limit of concern (LoC) is a trigger value for effect sizes in field experiments. Endpoints with effect sizes outside these limits should be scrutinized and might need further investigation. Notably, there is no assumption that exceeding a LoC would necessarily indicate a harm to the environment. The term used in EFSA (2010b) is LoC, but it is also known as the equivalence limit. If the confidence intervals for the estimated effects are within LoCs, this is considered a proof of equivalence (EFSA, 2010b) or proof of safety (Millard, 1987; Perry et al., 2009). The most common approach, which we will also follow in this paper, is two one‐sided tests (TOST) approach, where a two‐sided 90% confidence interval is compared to both lower and upper limits to establish equivalence with 95% confidence (Perry et al., 2009; Schuirmann, 1987).

LoCs for count data are typically defined for ratios of mean counts such as a twofold increase. It is not easy to set upper or lower limits for these ratios. The LoC should preferably be based on ecological expertise and, according to EFSA (2010b), “can be defined by e.g., literature data, modelling, existing knowledge and policy goals.” In absence of quantitative data for individual taxa, in this study basic LoCs were tentatively set to 0.5 (i.e., 50% decrease) and 2 (i.e., 100% increase).

A main problem with count data is the inherent increased variability at lower abundances resulting in less precise estimates of effect sizes (see, e.g., van der Voet & Goedhart, 2015) and in a limited power, as compared to more abundant species, for rejecting the null hypothesis of no difference or the nonequivalence null hypothesis (e.g., Legendre & Legendre, 1998; Perry, Rothery, Clark, Heard, & Hawes, 2003; van der Voet & Goedhart, 2015). This has often led researchers to omit low‐abundance data from their analyses (e.g., Prasifka et al., 2008). Rare taxa are generally perceived to be of minor concern for ecological functions that can also be performed by more abundant taxa (Lyons, Brigham, Traut, & Schwartz, 2005; Rosenfeld, 2002). Therefore, rare taxa are generally omitted from the analysis, but this raises the question of what criteria should be used for omitting a taxon. We therefore propose to use a flexible system of setting limits of concern, with less strict limits of concern for rare taxa, to account for the large statistical uncertainty of low counts. More variation at low abundances is just a statistical property and should therefore not be seen by ecologists as a reason for concern in itself. Thus, a flexible system of assigning LoCs for taxa with low abundance may be used to reflect the biological ranges of no concern. The system we propose in Section 2.2 employs a threshold abundance value below which scaling of the LoCs is applied.

## 2 METHODS

### 2.1 Methods of statistical analysis

In field studies for ERA of GMOs typically counts of various taxa are observed, sometimes supplemented with continuous non‐negative data and/or percentage data. Variability of insect abundance should measure proportional changes according to most ecologists (Gaston & McArdle, 1994). Observed counts are generally log‐transformed, typically after the addition of one to avoid taking the logarithm of zero, to achieve homogeneity of variance and some degree of independence from the mean abundance, at least for high abundance data, after which statistical methods based on the normal distribution, such as analysis of variance, are used. Alternatively, the squared root transform of counts is taken. However, such data transformations hamper the use of equivalence tests because it is not immediately clear how a confidence interval for the difference between the GMO and the CMP for the transformed data should be back‐transformed to the ratio scale. One approach is to calculate a so‐called generalized confidence interval for the ratio (Krishnamoorthy & Mathew, 2003), and this approach is outlined in Goedhart and van der Voet (2014). In other fields of ecological research, counts are statistically analyzed by log‐linear models which rely on distributions specific for count data such as the Poisson, the overdispersed Poisson (or quasi‐Poisson), and the negative binomial distribution (McCullagh & Nelder, 1989). Log‐linear models for ecological count data have been advocated for many years, see, for example, Sileshi (2006), Ver Hoef and Boveng (2006), O'Hara and Kotze (2010), Szöcs and Schäfer (2015), and Warton (2018). Such models provide a direct estimate of the log‐ratio of the means of the GMO and the CMP making equivalence testing straightforward. In a simulation study, Goedhart and van der Voet (2014) found that the transformation approach has good properties when it comes to difference testing but that generalized confidence intervals for the true ratio of the mean of the GMO and the CMP have poor coverage probabilities. The coverage probability of the log‐linear model employing the overdispersed Poisson distribution is generally satisfactory even when data are simulated according to other count distributions. Based on these simulations, statistical analysis according to the overdispersed Poisson model is recommended for equivalence testing based on count data. Szöcs and Schäfer (2015) also suggest to use the overdispersed Poisson model for count data in one‐way factorial experiments.

### 2.2 Adapted limits of concern for count data of nonabundant taxa

A flexible system of assigning LoCs for taxa with low abundance is proposed to reflect the ranges of no concern. Below a chosen limit abundance value, for example,* μ*_0_ = 10, it is proposed to apply a scaling to the LoCs for taxa. The scaling factor is $\sqrt{\mu_{0}/m}$, to be applied to the logarithms of the LoCs, in which *m* is the combined mean of the GMO and CMP. This implies that limits of concern become wider for lower abundances, corresponding to less concern at these low levels. Note that equivalently the scaled LoC equals the LoC raised to the power $\sqrt{\mu_{0}/m}$. For example, with basic LoCs at 0.5 and 2 and a threshold of *μ*_0_ = 10, the adapted LoCs are 0.38 and 2.7 for taxa with an abundance of 5 per plot, and 0.11 and 9.0 for taxa with an abundance of 1 per plot. The use of $\sqrt{1/m}$ in the scaling factor for the logarithms of the LoCs can be motivated by statistical large sample theory for the Poisson distribution. Suppose we have two samples each of size *n* from a Poisson distribution with means *μ*_1_ and *μ*_2_ respectively. The maximum‐likelihood estimator for the log‐ratio $\Delta =\log\left(\mu_{1}/\mu_{2}\right)$ is given by log(*X*_m_/*Y*_m_) in which *X*_m_ and *Y*_m_ are the respective sample means. Suppose that $\mu_{1}=\mu_{2}=\mu \mu_{0}$. The large sample variance of log(*X*_m_/*Y*_m_) then equals 2/(*nμ*). Consequently, the asymptotic standard error on the log‐ratio scale is proportional to $\sqrt{1/\mu }$ and the length of the confidence interval is thus also proportional to $\sqrt{1/\mu }$. It is then natural to use $\sqrt{1/m}$ as a scaling factor for the logarithm of the LoCs for means smaller than *μ*_0_ = 10. In a simulation study (Supporting Information Appendix S1), it was shown that the power of the equivalence test for the two‐sample case with the proposed adaptive limits of concern is approximately constant for $\mu \mu_{0}$.

When no single specimen is found for the GMO and/or the CMP, the resulting estimate for the ratio is zero, infinite, or not defined. Pragmatically, the ratio was then calculated with the zero average replaced by the lowest possible value, which is one over the number of replications. This ratio (without a confidence interval) is only displayed in case it falls outside the equivalence region.

### 2.3 Confidence intervals versus tests, graphical summaries

Often the final aim of an NTO study is implicitly framed as testing hypotheses about unintended differences. This is then presented as, for example, tables of means with indications of nonsignificant differences (e.g., Al‐Deeb & Wilde, 2003; Duan, Head, Jensen, & Reed, 2004). However, this way of presentation obscures the magnitude of the observed differences, the precision of these estimates and the criteria (limits of concern) against which the differences should be interpreted. More insight is provided by presenting the results as confidence intervals for the true effects, together with the LoCs.

Confidence intervals for effects and LoCs can be displayed for multiple endpoints together in a single graph. A background coloring may be applied to the area within the LoCs to indicate its meaning as equivalence area; that is, the observed data do not indicate concern under the specified criteria. On the other hand, no background coloring is applied to the area outside the LoCs, because in the proposed system the LoCs act as a trigger for further consideration, but values outside the LoCs do not necessarily indicate the presence of environmental harm.

A more general way of plotting allows a simultaneous display of endpoints measured at potentially very different scales. For this, the effect estimates and the corresponding confidence limits are scaled. The scaled dimensionless measure is called the LoC‐scaled difference (LoCSDIF) or, as it has been termed in related work (van der Voet, Goedhart, & Schmidt, 2017), the equivalence limit scaled difference (ELSD). For count data, if *Q* is the estimated ratio for GMO versus CMP, and if lower and upper LoCs are also expressed as ratios LoC_low_ and LoC_upp_ (which are assumed to be respectively below 1 and above 1, e.g., 0.5 and 2), the LoCSDIF is defined as follows

$$\text{LoCSDIF}=\left\{\begin{array}{c} \frac{\log(Q)}{-\log({\text{LoC}}_{\text{low}})}\;\text{if}\;Q<1\\ \frac{\log(Q)}{\log({\text{LoC}}_{\text{upp}})}\;\text{if}\;Q\geq 1 \end{array}\right.$$

For one‐sided problems, that is, when there is only one LoC, only the single expression with the specified LoC is used for all values of *Q*.

The LoCSDIF scale makes a distinction between increases and decreases in abundance (positive and negative effects). For an effective integration of concerns about both increases and decreases, we can also define the concern quotient CQ, which is a non‐negative score that expresses absence of concern for values up to 1:

$$\text{CQ}=\max\left[\frac{\log(Q)}{\log({\text{LoC}}_{\text{low}})},\frac{\log(Q)}{\log({\text{LoC}}_{\text{upp}})}\right]$$

For one‐sided tests, again only the expression with the relevant limit of concern is used and values smaller than 0, which express no concern, are replaced by 0.

**Figure 3:**
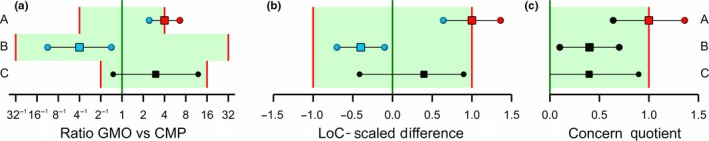
Graphical representation of a comparative analysis for hypothetical taxa A, B, and C. Point estimates and 90% confidence intervals for the ratio of the GMO versus the CMP (panel a) along with hypothetical limits of concern (red lines). Panel b shows the same interval as LoC‐scaled differences. Panel c shows the same interval as Concern Quotients. Points outside the LoCs are colored red, and points inside the LoCs for statistically significant differences are colored blue

A hypothetical example of plots on the ratio scale (*Q*), the LoC‐scaled difference scale (LoCSDIF)*,* and the concern quotient scale (CQ) are shown in Figure 3, with unequal limits of concern for three taxa. In plot (a), the hypothetical Taxon A and Taxon B are seen to be significantly different from zero because their intervals do not overlap the vertical equality line at a ratio of 1. But the fourfold decrease for Taxon B is not considered a concern, whereas the fourfold increase for Taxon A, colored red, is a concern. In a similar way, the threefold increase for Taxon C is not considered a concern. The ordering of concerns is easier seen in plots (b) and (c) for the LoCSDIF and CQ scale. Note that for Taxon C scaling on the right is done with the upper LoC which is 16, while scaling on the left employs the lower LoC which is 0.5. Real examples of plots showing both types of graphical representation are given in Section 3.2.

### 2.4 Summarizing over different dimensions

In the design phase of the experiment, the proposed protocol requires preparation of a hierarchical tree of endpoints (Section 1.1). In this section, the general approach for the analysis of equivalence when following this tree is outlined. For an illustration, see Section 3.

**Figure 4:**
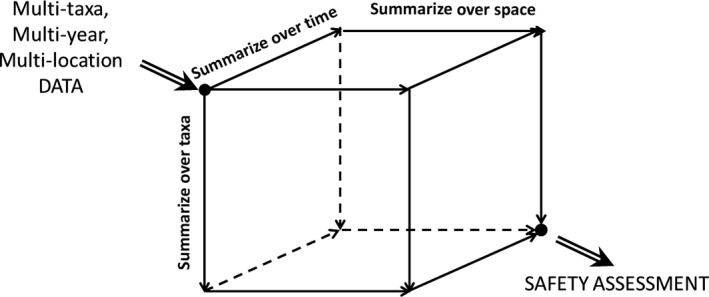
Possible routes for integration over space, time, and taxa in environmental risk assessment

A typical ERA study will account for biogeographical variation by counting many taxa at multiple sites during multiple years. The data selected for analysis may therefore have different taxa for different space–time combinations. In general, there are multiple ways how data can be integrated over different sites, different years, and different taxa to obtain an overall conclusion for the safety assessment (see Figure 4).

As can be seen in Figure 4, there are six possible ways to summarize conclusions given by the sequences: (time, space, taxa), (time, taxa, space), (space, time, taxa), (space, taxa, time), (taxa, time, space), and (taxa, space, time).

For each integration step, there are in principle three method types for summarizing:

Method type 1: Preprocessing of the data, for example, summing counts over taxa, summing counts over time, or the calculation of a biodiversity index to summarize over taxa;
Method type 2: Joint data analysis resulting in estimates of effects—this is applicable for summarizing over time or over space, but only when the same taxa are addressed;
Method type 3: Multicriteria decision analysis (MCDA) applied to estimates of effects which is applicable for all forms of summarizing.

These three method types are ordered in the sense that, for example, Method type 2 can only be followed by Method type 2 or 3 in the next integration step. We further distinguish between various forms of data analysis. In the hierarchies A and B given in Figure 2, the statistical analysis (SA) estimates effects from data without further integration: In hierarchy A, data per taxon were summarized to an effect per taxon, while in hierarchy B data per time points were summarized to an effect per time points per taxon. In hierarchy B, however, the statistical analysis could also, in one go, summarize over time points giving a single effect per taxon. Such an analysis will be termed a statistical hierarchical analysis (SHA), because it estimates effects for a higher level in the hierarchy.

Individually estimated effects, for example, for several taxa, can be combined in a single effect and a corresponding confidence interval by means of a statistical meta‐analysis (SMA). This provides an objective way of combining information from separate effects, while taking into account the different standard errors for the individual effects, see, for example, Hardy and Thompson (1996). There are basically two versions of meta‐analysis. The “fixed” version assumes that estimated effects have a common mean and individually known variances. The overall effect is then simply the weighted average of the individual effects, in which the individual variances are used as weights. The “random” version on the other hand allows for heterogeneity of the individual effects by introducing a between individuals component of variance. Residual maximum likelihood (REML) can then be used to estimate the overall effect and to provide a confidence interval for the overall effect. In ERA, we may expect heterogeneous effects for individual taxa, and therefore, we applied REML to the estimated log‐ratio effects. Note that a meta‐analysis implicitly assumes statistical independence of the individual effects. This might be an unrealistic assumption when combining information for different taxa within the same experiment. Also note that SMA implicitly assumes that negative effects, for example, for a taxon, can be compensated by positive effects for another taxon.

In the equivalence analysis (EA), a statistical proof of equivalence is established if the confidence interval does not exceed any limit of concern. According to the terminology of EFSA (2010a), equivalence is more likely than not if the estimated effect (point estimate) falls between the lower and upper limits.

In summarizing different equivalence analyses, the simplest but most stringent option for a proof of safety is to require that all confidence intervals are within their LoCs (EA_all,interval_). An alternative and less stringent option, related to the notion of “equivalence more likely than not,” is to require that all point estimates of the effects are within their LoCs (EA_all,point_). Other options are based on averaging point estimates on the LoCSDIF or CQ scale, and compare this average to the scaled limits of concern, which are −1 and 1 for LoCsDIF, or 1 for CQ (EA_av,LoCSDIF_ or EA_av,CQ_).

Table 1 summarizes the different possible steps in building a hierarchy for the analysis of observed data. Note that summarizing by means of SHA, SMA, or EA_av_ implies that we are interested in an average effect. In contrast, EA_all_ considers all individual effects on their own.

**Table 1:** Elements of the hierarchy for data analysis and integration of equivalence

Element	Explanation
Method type 1: Data preprocessing	
SUM	Summing the data. For example, summing counts of a taxon over different points in time, or summing counts of taxa within the same functional group to give a single count for the functional group
INDEX	Calculation of a diversity index, for example, the Shannon–Wiener or Simpson index (Lazebnik et al., 2017)
Method type 2: Statistical analysis	
SA	Statistical Analysis of data resulting in estimated effects at the same level of the hierarchy, that is, without integration of other levels in the hierarchy. For example, estimation of the effect for a single taxon per time points.
SHA	Statistical hierarchical analysis of data resulting in estimated effects at a higher level of the hierarchy, that is, including integration of other levels in the hierarchy. For example, estimation of the effect for a single taxon summarized over time points.
SMA	Statistical meta‐analysis which combines individual effects into a single combined effect. For example, combining effects for taxa within the same functional group to give a single effect for the functional group, or combining effect for individual environments to give a single effect across environments.
Method type 3: Equivalence analysis (multicriteria decision analysis)	
EA_all_	Equivalence analysis of estimated effects in which all estimated effects should meet the equivalence criterion. This step can be present several times, for example when moving from (a) equivalence per functional group per year per site to (b) equivalence per year per site to (c) equivalence per site to (d) overall equivalence. This step can be applied using confidence intervals (EA_all,interval_) for a statistical proof of safety or using point estimates (EA_all,point_) for an assessment whether equivalence is more likely than not
EA_av_	Equivalence analysis of estimated effects in which the average of estimated effects should meet the equivalence criterion, where effects are first rescaled to the LoCSDIF scale (EL_av,LoCSDIF_) or the CQ scale (EA_av,CQ_). This step can also be present several times

Note

An element can only be followed by an element which has an equal or higher numbered method type.

### 2.5 A protocol for the statistical equivalence analysis of NTO effects

In this section, we present a protocol for the statistical analysis of data from ERA field trials. In principle, the methods of statistical analysis should have been decided at the planning stage of the experiment, but it may be needed to update the methods based on the context or unexpected findings.

#### 2.5.1 General

1. When the experiment was designed, a *list of NTO endpoints* should have been prepared. This will typically be organized in a hierarchy, see, for example, Figure 1. This list may include taxa which may or may not be present under the conditions of the experiment. If necessary, update the list with any unexpected findings. Motivate any change to the initial list of endpoints and its hierarchy at the end of the experiment, but before the statistical analysis.
2. Already at the design stage, a *logical tree for the analysis* shows should have been prepared, specifying how *data* will be preprocessed (*data preprocessing steps*, Method type 1), how *effects* will be estimated from the data by statistical analysis (*statistical analysis steps*, Method type 2), and how conclusions on *equivalence* will follow from the set of estimated effects and the limits of concern which should also be specified at the design stage of the experiment (*equivalence analysis steps*, Method type 3). The branches of the trees may have different schemes for the subtrees; for example, Carabidae may be summed over different point in time while for Collembola a statistical analysis is envisaged for each individual point in time. In general, many different trees will be possible; therefore, the chosen tree should be motivated. In the analysis stage, check and if necessary update the logical tree for the analysis of all observed endpoints. Motivate any change.
  - For count data, a typical way of *preprocessing* the data is to *sum* over primary levels, for example, over individual time points to obtain year totals, or over individual taxa to obtain totals for functional groups.
  - Indicate the *nature of the statistical analysis steps* in the logical tree as being a statistical analysis (SA, where the effects are calculated at the same level as the data), a statistical hierarchical analysis (SHA, where the data are at a lower level of integration than the estimated effects) or a statistical meta‐analysis (SMA, where effect estimates of a previous analysis are integrated to a higher level). More guidance is provided in Section 2.5.2 (SA) and Section 2.5.3 (SHA and SMA).
  - Indicate the *nature of the equivalence analysis integration steps* in the logical tree as requiring equivalence conclusion to be valid for all members (EA_all_) or as allowing members to compensate for each other by averaging of LoC‐scaled differences or concern quotients (EA_av_).
3. Present the results of the statistical analyses by graphical summaries of estimated effects and, if deemed useful, of LoC‐scaled differences or concern quotients CQ (Section 2.5.4).

#### 2.5.2 Statistical analysis of single endpoints

The basic approach is to calculate estimates and 90% confidence intervals for effects (GMO vs. CMP differences, expressed on an appropriate scale), and then compare these to the (possibly provisional) limits of concern which were specified during the design of the experiment.

1. The method of statistical analysis depends on the type of endpoint. For continuous endpoints with necessarily positive values, it is recommended to perform an analysis on the log‐transformed data. For discrete endpoints such as count data and fraction data, it is recommended to perform an analysis on the original scale using an appropriate statistical distribution and link function.
2. Analyze the transformed data by linear models: ANOVA if the design is balanced, or by regression or a mixed model (REML in case there are additional random effects) if it is not.
3. Analyze the count data by generalized linear models (GLM) or by generalized linear mixed models (GLMM) in case there are additional random effects in the model. Allow for overdispersion in counts whenever necessary.
4. Check whether statistical assumptions are reasonable, for example, as follows:
  - Outliers: check data points with large standardized residuals. Compare analyses with and without such data points.
  - Check a normal probability plot of the standardized residuals for large deviations from linearity.
  - A plot of standardized residuals versus fitted values can be used to check if there is heteroscedasticity.
5. If statistical assumptions are not met, then an ad hoc strategy will have to be followed. For example, another variance function might be more appropriate or nonparametric tests may be used. This protocol further assumes that the model fits sufficiently well.
6. Extract the estimated difference between the GMO and CMP from the statistical model, for example, the log‐ratio for count data, and calculate a two‐sided 90% interval. Display the confidence interval in a graph along with the LoCs. For visual display, it is recommended to calculate and display both confidence limits, even if there is only one LoC.

#### 2.5.3 Statistical analysis integrating multiple endpoints

1. The use of SHA or SMA is only logical if LoCs are defined for the integrated output or if LoCs are equal for all individual endpoints.
2. Integration over multiple endpoints may be automatically performed in a statistical hierarchical analysis (SHA) model as described in Section 2.5.2. Perform a statistical meta‐analysis (SMA) if requested by the logical tree for analysis. For this, consider the estimated effects with their standard errors (at an appropriate scale, e.g., the log scale) as input for the meta‐analysis.
3. From the analysis, construct an estimate and a 90% confidence interval for the overall effect.

#### 2.5.4 Graphical representation of effects

1. For each endpoint, *plot point estimates and 90% confidence intervals of estimated effects*, together with lines for the equality ratio 1 and for the LoCs. In most cases, plots on a logarithmic scale are advised. The 90% limits of the interval represent a 5% significance level for equivalence testing in a two one‐sided tests (TOST) approach.
2. Visualize possible groupings in the hierarchy which are of interest as specified in the logical tree for analysis.
3. Compare the intervals to the LoCs to obtain *conclusions regarding equivalence* of the GMO and the CMP. Use different symbols or colors for confidence intervals that do not fall within the LoCs.
4. If of interest, compare the intervals to zero to obtain conclusions regarding the *statistical significance of the difference* between the GMO and the CMP. Note that this implicitly employs a significance level of 10% for a two‐sided difference test. Use different symbols or colors for significant differences.
5. Optionally, confidence intervals can be displayed on the LoC‐scaled difference (LoCsDIF) scale or on the concern quotient (CQ) scale. This possibly allows an easier comparison in case limits of concern are not the same for various endpoints.

## 3 CASE STUDY: NONTARGET ORGANISMS IN POTATO FIELD TRIALS

Field trials with potato were performed in Ireland and the Netherlands in 2013 and 2014 (Kessel et al., 2018; Lazebnik, Dicke, Braak, & Loon, 2017) and are summarized in Table 2. The main purpose was to compare a cisgenically modified late blight resistant potato line, called A15‐13 (GMO), with its conventional comparator cultivar Désirée (CMP). Another conventional variety, SarpoMira, was also included in the trial. Both conventional varieties and the cisgenic potato genotype were subjected to three late blight control strategies: (a) Weekly spraying with fungicides which is common practice in the Netherlands and Ireland, (b) no spraying, and (c) spraying according to an advanced level of integrated pest management (IPM2.0, Kessel et al., 2018). In the sequel, *Genotype* denotes the three genotypes, *Spraying* denotes the late blight control strategy, and *Treatment* denotes the nine combinations of *Genotype* and *Spraying*. The main interest for safety assessment was the comparison of the GMO with the IPM2.0 control strategy with the CMP when weekly spaying is applied. Completely randomized block designs were employed, and a separate randomization was carried out for each of the four experiments. The number of replications was six in Ireland and seven in the Netherlands. For the purpose of assessing unintended effects on NTOs, pitfall traps were placed in every plot for one week and emptied three times during each trial, with about four weeks between two trapping sessions. The scope of the assessment was restricted to arthropods. Arthropods were identified and counted in each pitfall trap. Taxa were grouped into six functional groups: Predators, Detritivores, Parasitoids, Fungivores, Herbivores, Hyperparasitoids, and a seventh group “Unknown” for remaining taxa. Statistical calculations were performed with GenStat (VSN International, 2017).

**Table 2:** Experiments comparing three potato genotypes in two countries and 2 years, showing the number of plots (replicates, blocks) for each of the nine Treatments (combinations of Genotype and Spraying)

Number of plots (blocks)		Ireland		Netherlands	
Genotype	Spraying	2013	2014	2013	2014
A15‐13 (GMO)	Weekly	6	6	7	7
A15‐13 (GMO)	No spraying	6	6	7	7
A15‐13 (GMO)	IPM2.0	6	6	7	7
Désirée (CMP)	Weekly	6	6	7	7
Désirée (CMP)	No spraying	6	6	7	7
Désirée (CMP)	IPM2.0	6	6	7	7
SarpoMira	Weekly	6	6	7	7
SarpoMira	No spraying	6	6	7	7
SarpoMira	IPM2.0	6	6	7	7

Note

The comparison of main interest for safety assessment is shown in the two rows with a gray background.

### 3.1 Hierarchies to analyze NTOs in the four potato trials

**Figure 5:**
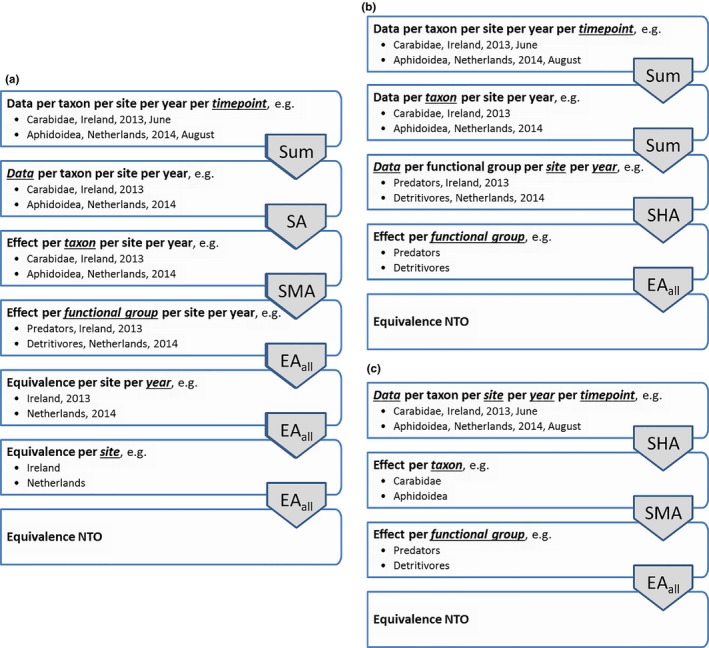
Examples of logical trees for the analysis of NTO data for potato field trials in Ireland and the Netherlands in 2013 and 2014. EA_all_: equivalence analysis requiring all effects (confidence intervals or point estimates in two variants of the procedure) to be within their limits; SA: statistical analysis; SHA: statistical hierarchical analysis; SMA: statistical meta‐analysis; Sum: summation of data

Figure 5 shows three examples of hierarchies for analyzing the NTO data. Details of the steps depicted in Figure 5 and their implicit assumptions are detailed below. For hierarchy A in Figure 5, the steps were as follows:

A.1 SUM: The first step in hierarchy A is to sum the count data for each individual taxon over the three time points which results in a single count for every taxon for each plot per site per year. This was done because not enough power was expected at single time points especially for the less abundant taxa. Summing disregards interactions with time points within experiments.
A.2 SA: Counts of single taxa within experiments are statistically analyzed to give effects for each taxon per site per year. This enables us to inspect the effect for every single taxon per site per year. This is useful when decisions regarding individual taxa need to be made for different experimental conditions.
A.3 SMA: Effects for taxa within the same functional group are combined per site per year using a meta‐analysis. This assumes that a negative effect for a taxon can be compensated by a positive effect for another taxon within the same functional group. Effects with large standard errors, for example, due to low abundances, have a lower weight in the meta‐analysis. This implies that the overall effect is dominated by effects with small standard errors and these are generally taxa with high abundances.
A.4 EA_all_: The combined effects for the functional groups are first evaluated for each combination of sites and years, both using confidence intervals for a proof of safety approach and using the point estimates to establish whether equivalence is more likely than not. This would give a single result for each site for each year, which could be used for site‐ and year‐specific decisions.
A.5 EA_all_: These combined CQs are then assessed over years for each site.
A.6 EA_all_: And finally, the CQs for sites are combined into a single judgment.

For hierarchy B, in Figure 5, the steps are as follows:

B.1 SHA: This is identical to step A1 described above.
B.2 SUM: The taxa are further summed to form counts for functional groups. This implicitly assumes that individuals of different species within the same functional group are equally valuable. It also presumes that there is no interest in individual taxa.
B.3 SHA: A statistical hierarchical analysis is performed to estimate the effect for each functional group while averaging over years and sites (e.g., summarizing results at European scale). This implicitly assumes that there is only interest in a cross‐environment estimate of effects and that negative effects in one environment can be compensated by positive effects in another environment. It also assumes that decisions based on just a single experiment (possibly involving national decisions) are not of interest.
B.4 EA_all_: The effects obtained in the previous step are assessed over functional groups

For hierarchy C, in Figure 5, the steps are as follows:

C.1 SHA: A statistical hierarchical analysis is performed to estimate the effect for each taxon while averaging over time points, years, and sites. This implicitly assumes there is only interest in a cross‐environment estimate of effects and that negative effects in one environment can be compensated by positive effects in another environment. It also assumes that national decisions are not of interest.
C.2 SMA: effects for taxa within the same functional group are combined. This assumes that a negative effect for a taxon can be compensated by a positive effect for another taxon within the same functional group. Effects with large standard errors, for example, due to low abundances, have a lower weight in the meta‐analysis. This implies that the overall effect is dominated by effects with small standard errors and these are generally taxa with high abundances.
C.3 EA_all_: The effects obtained in the previous step are assessed over functional groups.

Still other hierarchies are possible, such as the approach based on first summarizing over taxa by calculating diversity indices, which has been described for this specific case study in Lazebnik et al. (2017).

### 3.2 Safety assessment for NTOs in the potato trials

The analysis according to hierarchy A provides the most detail and is presented below. In step A1, the counts for each taxon are summed over the time points. Data from two pitfall traps in the Irish trial in 2013 were missing at the second time points. To enable summing over time points, these missing counts were imputed using the log‐linear model “*Block* + *Treatment*” for the time points in question. The same was done for a single missing pitfall at the second time points in the Dutch trial in 2014. For the Dutch 2013 trial, 13 out of 63 traps were missing for the first and third time points. Therefore, for this trial the first and third time points were discarded.

#### 3.2.1 Estimation of the difference between GMO and CMP in the potato trials

In step A2, each taxon was statistically analyzed separately for each experiment. The analysis accounted for differences between blocks and resulted in an estimate of the log‐ratio for the GMO versus CMP comparison. In principle, the interest was in a comparison between two of the nine treatments, namely GMO with IPM2.0 versus CMP with weekly spraying (see gray rows in Table 2). However, when there is no interaction between *Genotype* and the *Spraying* treatments, the effective level of replication can be increased by a factor of three by investigating the difference between the GMO and CMP averaged over the three control strategies. This can be accomplished by fitting the main effects model “*Block* + *Spraying* + *Genotype.*” It is customary to use this main effects model in case the interaction is not significant. However, the interaction between *Genotype* and *Spraying* has four degrees of freedom and also involves the additional variety SarpoMira which is of no interest for the main comparison between the GMO and CMP. So it is possible that an interaction between *Spraying* and the GMO/CMP is swamped by complete absence of an interaction with SarpoMira or the other way around. This problem can be settled by excluding the additional variety from significance testing of the interaction. The remaining interaction is then between GMO/CMP on the one hand and *Spraying* on the other hand. Moreover, the *Spraying* treatment “None” can be fully responsible for the remaining interaction in which case we would like to compare the GMO and CMP averaged over the two *Spraying* treatments “IPM2.0” and “Weekly.” These considerations were formalized in the following procedure:

1. Test for the interaction between GMO/CMP and *Spraying* (with three levels) which has two degrees of freedom. In case this interaction is not significant, compare the GMO and CMP averaged over the three *Spraying* levels. Otherwise go to 2.
2. Test for the interaction between GMO/CMP and the *Spraying* levels Weekly and IPM2.0; this interaction has one degree of freedom. In case this interaction is not significant, compare the GMO and CMP averaged over the two *Spraying* levels Weekly and IPM2.0. Otherwise go to 3.
3. Fit the full model “*Block* + *Treatment*” and compare GMO‐IPM2.0 versus CMP‐Weekly.

In the sequel, the GMO and CMP treatments will refer to the means averaged over three, two, or one level(s) depending on the outcome of the interaction tests.

#### 3.2.2 Effects per taxon per site per year

When either the GMO or the CMP treatment has a zero mean count, the log‐ratio, that is, Δ = log(*Q*), would be estimated as plus or minus infinity. In these situations, the zero mean count was pragmatically replaced by the smallest positive mean possible, that is, one divided by the number of replicates. In most cases, the zero count was combined with a low count for the other genotype, resulting in a ratio not far from one. In these cases, a confidence interval was not computed, and in the proposed graphical summaries, only calculated ratios outside the LoCs were signaled.

**Figure 6:**
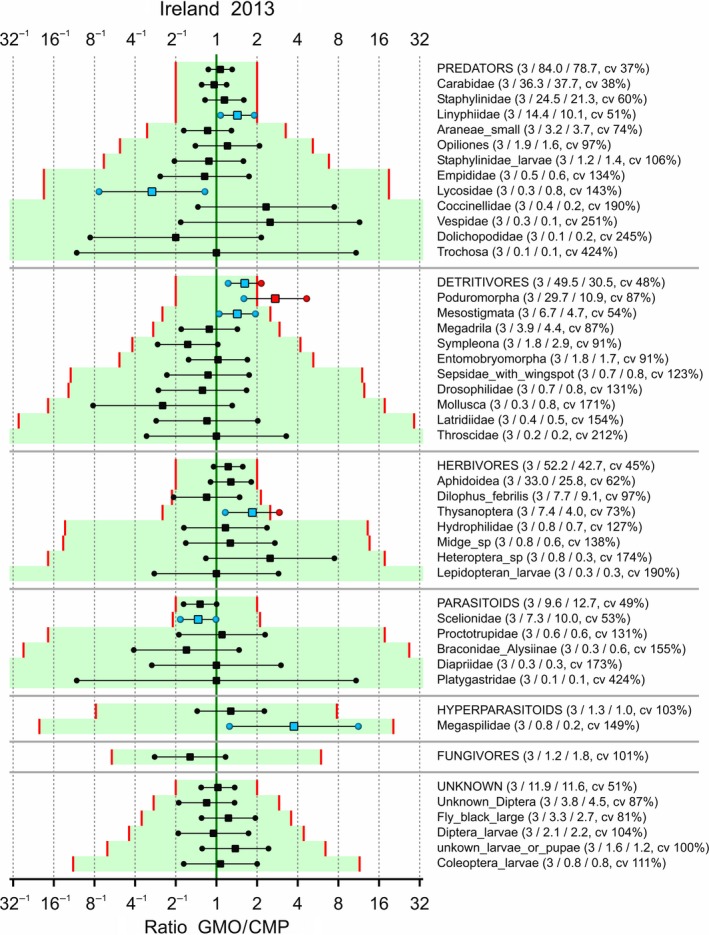
Arthropods in potato trial in Ireland 2013. 90% confidence intervals for the ratio between GMO and CMP averaged over control strategies if possible. Added in parentheses are the number of control strategies over which is averaged, the means for the GMO and CMP, and the coefficient of variation (cv). Limits of concern (red lines) equal 0.5 and 2, and log(LoC) is scaled by $\sqrt{10/m}$ for combined means *m* lower than 10. Points outside the LoCs are colored red, and points inside the LoCs for statistically significant differences are colored blue

**Figure 7:**
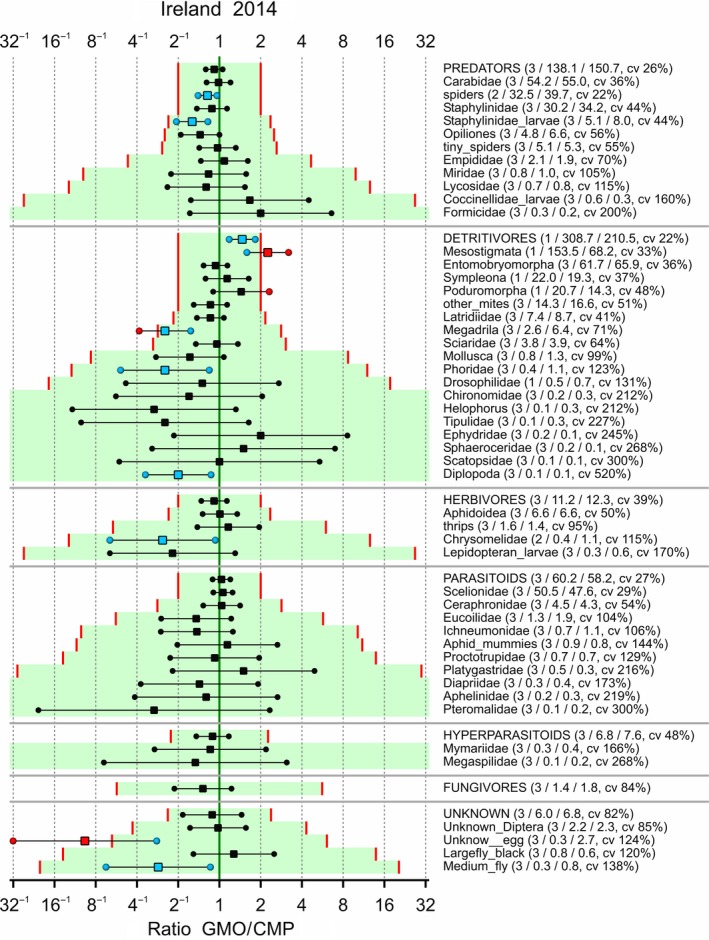
Arthropods in potato trial in Ireland 2014. 90% confidence intervals for the ratio between GMO and CMP averaged over control strategies if possible. Added in parentheses are the number of control strategies over which is averaged, the means for the GMO and CMP, and the coefficient of variation (cv). Limits of concern (red lines) equal 0.5 and 2, and log(LoC) is scaled by $\sqrt{10/m}$ for combined means *m* lower than 10. Points outside the LoCs are colored red, and points inside the LoCs for statistically significant differences are colored blue

**Figure 8:**
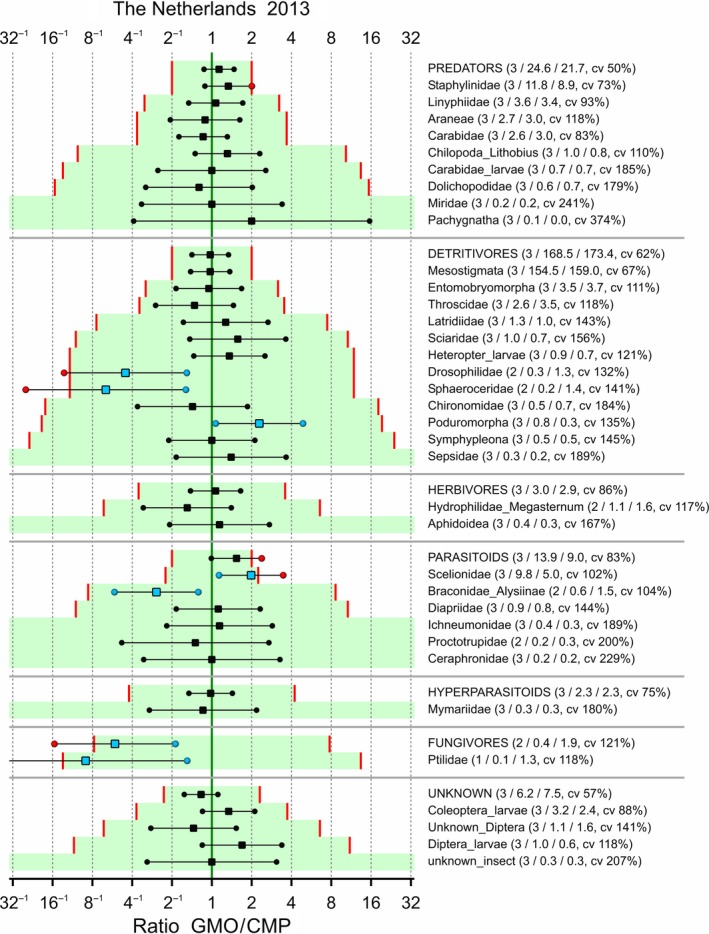
Arthropods in potato trial in the Netherlands 2013. 90% confidence intervals for the ratio between GMO and CMP averaged over control strategies if possible. Added in parentheses are the number of control strategies over which is averaged, the means for the GMO and CMP, and the coefficient of variation (cv). Limits of concern (red lines) equal 0.5 and 2, and log(LoC) is scaled by $\sqrt{10/m}$ for combined means *m* lower than 10. Points outside the LoCs are colored red, and points inside the LoCs for statistically significant differences are colored blue

**Figure 9:**
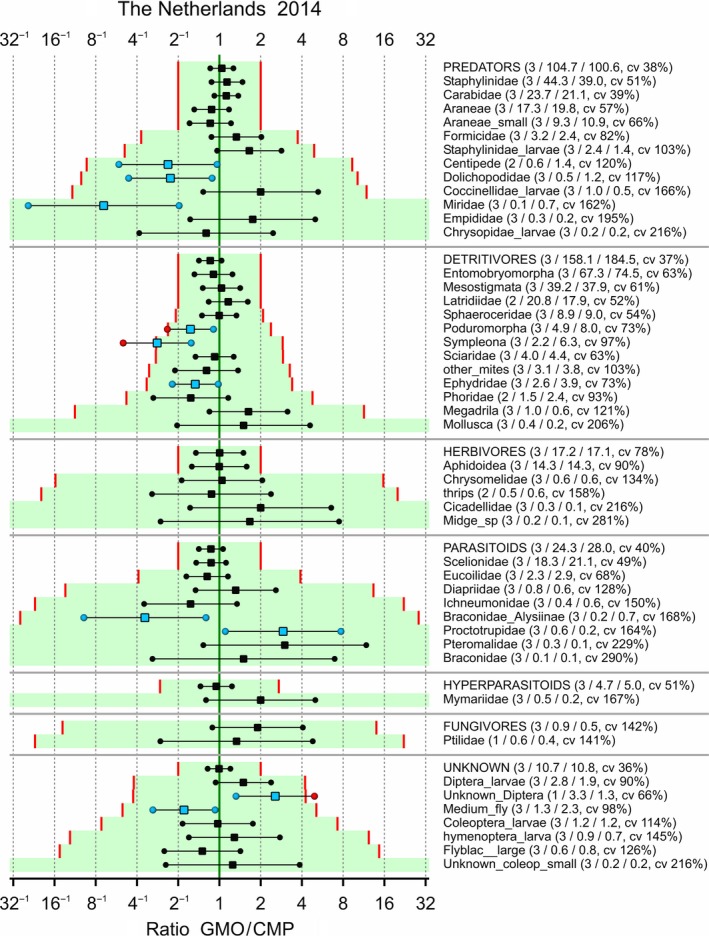
Arthropods in potato trial in the Netherlands 2014. 90% confidence intervals for the ratio between GMO and CMP averaged over control strategies if possible. Added in parentheses are the number of control strategies over which is averaged, the means for the GMO and CMP, and the coefficient of variation (cv). Limits of concern (red lines) equal 0.5 and 2, and log(LoC) is scaled by $\sqrt{10/m}$ for combined means *m* lower than 10. Points outside the LoCs are colored red, and points inside the LoCs for statistically significant differences are colored blue

Limits of concern were tentatively set to 0.5 and 2 for each taxon, and the LoCs were adapted such that the logarithm of LoC was multiplied by $\sqrt{10/m}$ whenever the combined mean *m* of the GMO and CMP was below 10. The confidence interval for each effect, along with the associated LoCs, is given in Figures 6, 7, 8, 9. Note that a confidence interval for each functional group is also given; this is for the sum over the taxa within each group which was part of the analysis according to hierarchy B. Also note that the ordering of the species within each functional group is according to the adapted limit of concern because this is visually more attractive. This has the drawback that the ordering in the figures is different. There are two abundant species with an estimated effect which is outside the tentative LoCs: Poduromorpha in Ireland‐2013 and Mesostigmata in Ireland‐2014. Most intervals fall completely within the LoCs. Supporting Information Appendix S2 displays the same figures on the LoCSDIF and CQ scales. Obviously, all point estimates outside the limits of concern in Figures 6, 7, 8, 9 correspond to concern quotients >1.

#### 3.2.3 Integrated analysis

**Figure 10:**
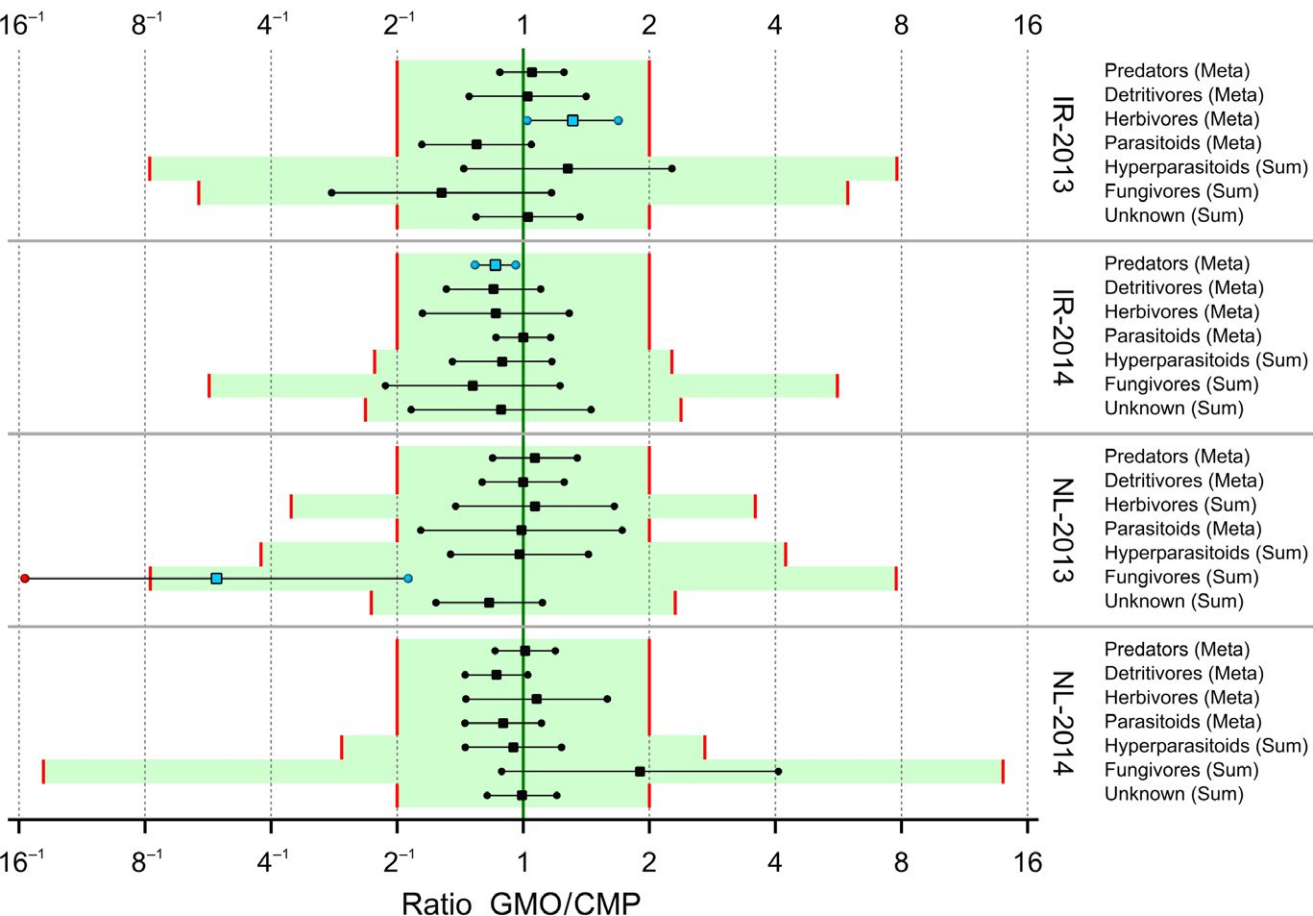
90% confidence interval resulting from a meta‐analysis for functional groups for arthropods data in potato field trials. Meta‐analysis is only performed for those functional groups with four or more taxa. For other groups, the interval for the sum counts is given. Points outside the LoCs (red lines) are colored red, and points inside the LoCs for statistically significant differences are colored blue

**Figure 11:**
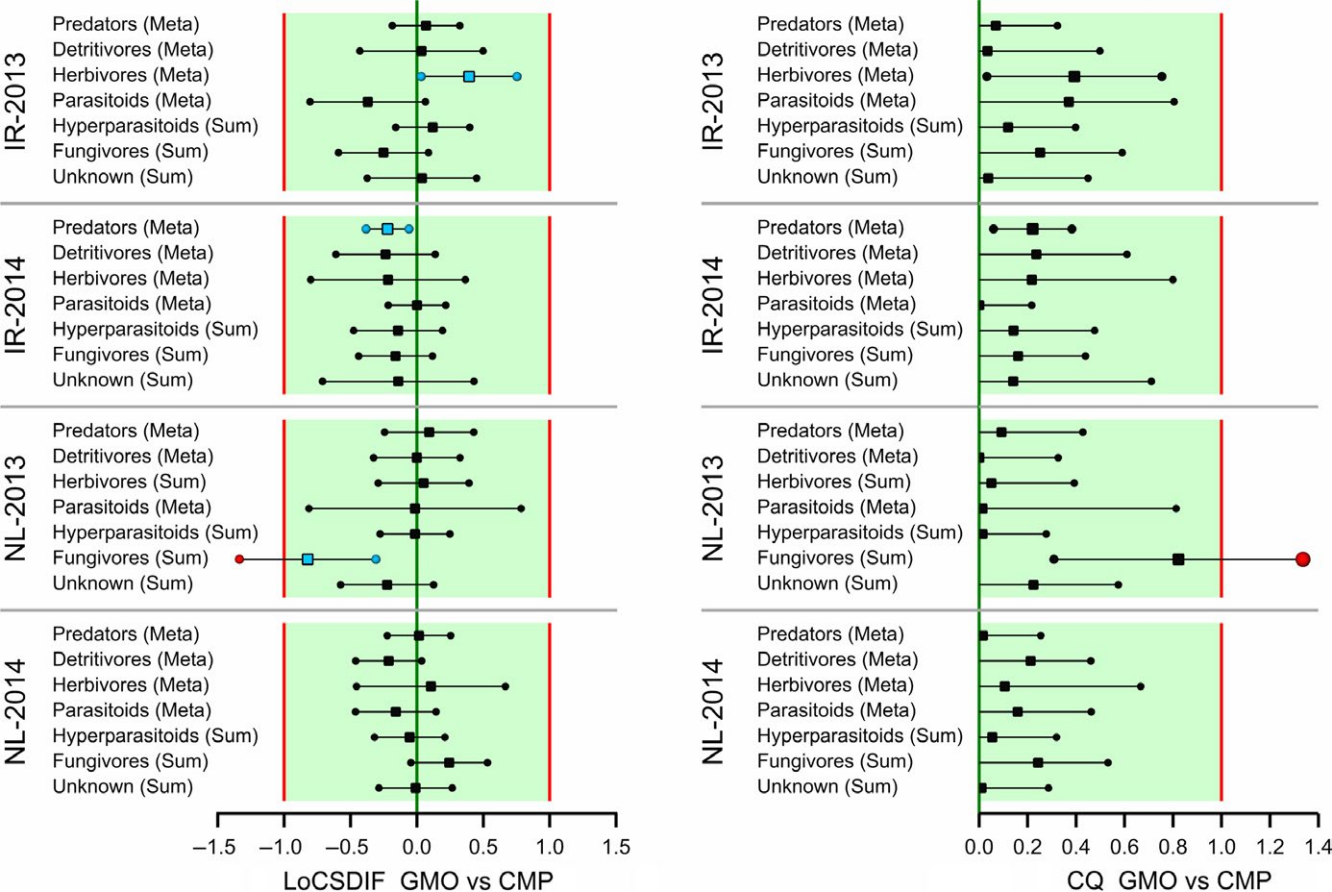
Confidence intervals for GMO versus CMP differences per functional group expressed as LoC‐scaled differences (LoCSDIF, left) and as Concern Quotients (CQ, right). The green areas indicate equivalence or no concern. Points outside [−1,+1] (red lines) are colored red, and points inside the LoCs for statistically significant differences are colored blue.

Step A3 involves a meta‐analysis for each functional group for each site/year combination. This is not useful when there are only a few taxa within a functional group. The meta‐analysis was therefore only carried out for those functional groups with four or more taxa. In case the functional group has three or fewer species, the estimated effect for the sum was taken. This was the case for Hyperparasitoids and Fungivores in all four experiments and Herbivores in the Netherlands 2013. Limits of concern for estimated overall effect for the meta‐analysis were again tentatively set to 0.5 and 2 and for the estimated effect for the sum as before. Confidence intervals are given in Figure 10 for the ratios and in Figure 11 for the LoCSDIF and CQ scales. All ratio intervals are within the LoCs except for the Fungivores interval for the trial in the Netherlands (NL) in 2013. Note that this involves very few individuals (0.4 for the GMO vs. 1.9 for the CMP) and also note that in the NL‐2014 trial the estimated effect for Fungivores has the opposite sign.

In step A4, an equivalence analysis (EA_all_) separately per site and year reveals that equivalence is established for IR‐2013, IR‐2014, and NL‐2014, but not for NL‐2013 due to the outlying Fungivores. Further integration in step A5 results in equivalence for Ireland but not for the Netherlands, and in step A6 no overall equivalence. In case EA_all_ is based on equivalence more likely than not, which only requires that the estimated effect is within the limits of concern, than all integration steps result in equivalence. The concern quotient at any of the steps A4, A5, or A6 is calculated as CQ = 0.8 with an upper confidence limit of 1.3 (Figure 11). The final result of the assessment is that the full data set of entomological counts across sites and years has thus been reduced by the proposed method to just one functional group at one site and one year (Fungivores, with reduced counts in NL‐2013), which should be inspected for further interpretation. We note again that this case is not labeled as causing any harm, but only as a trigger for further inspection.

## 4 DISCUSSION

The definition of appropriate limits of concern for ecological endpoints is a fundamental requirement to evaluate equivalence between treatments during ERA. The importance of setting appropriate limits of concern has been recognized by EFSA (EFSA, 2010b). However, there is some ambiguity about the intended meaning of this concept. On the one hand, LoCs are defined as “the minimum ecological effects that are deemed biologically relevant and that are deemed of sufficient magnitude to cause harm” (EFSA, 2010b, Glossary, p. 110). On the other hand, it is stated, in the general section on problem formulation, that:

… for each measurement endpoint, the level of environmental protection to be preserved is expressed through the setting of ‘limits of concern’ which may take one of two forms. For studies in the environment(s) that are controlled […] the limits of concern will usually be trigger values which, if exceeded, will either lead to conclusions on risks or the need for further assessment in receiving environment(s). For field studies, the limits of concern will reflect more directly the minimum effect that is considered to potentially lead to harm […]. If these limits are exceeded, then detailed quantitative modelling of exposure may be required to scale up effects at the field level both temporally and spatially (EFSA, 2010b, p. 15).

In this quote, two study types are identified: controlled studies (semifield trials, e.g., using cages in the field) and field studies. Despite some difference in wording, in both cases LoC is functioning as a trigger value for further attention. The words “need for further assessment” and “potentially” make clear that exceeding the LoC does not necessarily indicates a harm.

We therefore see, even in one important document, two different definitions of LoC:

1. Toxicity limits, that is, context‐dependent concepts indicating limits of harm to the environment, for example, extinction of a population; and
2. Equivalence limits, that is, pragmatic trigger values for further assessment after data analysis.

Toxicity limits are useful concepts, but require further ecological or toxicological modeling to quantify, which often will be very difficult to perform. Equivalence limits can be based on past experience and expert opinion, where exceeding limits is not indicating harm to the environment. In the current paper, we have used limit of concern (LoC) in the second meaning.

The LoC values 0.5 and 2 used in this paper are provisional, and open for revision where additional evidence might become available. Therefore, all results which depend on these LoCs (such as all equivalence test results) should be seen as the results of a scenario study using these provisional LoCs. Alternative scenarios can be considered if other appropriate LoC values would be proposed. Note that LoCs could in principle be defined separately for each endpoint, for example, based on population dynamics data; therefore, the choice of the same LoCs for all endpoints should not be read as a general suggestion.

Smaller counts are more variable by their very nature resulting in less frequent rejection of the nonequivalence hypothesis, that is, less frequent establishment of equivalence. Therefore, we propose to scale the LoCs for mean counts smaller than a threshold value *μ*_0_ resulting in a similar power to reject the nonequivalence hypothesis for all mean counts smaller than *μ*_0_. Although this is an ad hoc procedure, it does prevent triggering further research just because of statistical properties of count distributions with small means. In the case study, we employed *μ*_0_ = 10 without any justification. A prospective power analysis, for example, with different count distributions, could be employed to support other values. Goedhart et al. (2014) provide a framework for such a power analysis by means of a simulation tool.

In the four combinations of location and year, the observed taxa were not all the same (Figures 6, 7, 8, 9). For analyses integrated over time and space, it is therefore essential to group the data at the functional group level first.

A statistical analysis is often performed on only part of the data, for example, endpoint by endpoint, and results in estimated effects, that is, differences between GMO and CMP at an appropriate scale (often the log scale), as in Figures 6, 7, 8, 9, 10. These effects, and their confidence limits, can be standardized by scaling to a no‐concern yardstick, which represents a minimum limit of potential biological relevance, that is, the limit of concern (LoC). The resulting LoCSDIF scale, as in Figure 11, has the (visual) advantage that all endpoints have a common scale and that for all endpoints equivalence is established whenever the confidence interval fully lies in the interval (−1,1). The LoCSDIF scale can be further integrated in the Concern Quotient CQ scale, which does not differentiate between positive and negative differences. In the example analysis, the final result (Figure 11) was an estimated CQ of 0.8, with an upper confidence limit of 1.3 due to the decreased count of fungicides in one of the four trials and the use of the stringent EA_all_ method. Note that LoC_low_ and LoC_upp_ do both correspond to CQ = 1, that is, the threshold for concern.

For integration over time, space and/or endpoints some form of MCDA are commonly needed. In this paper, the assessment of equivalence was done by checking whether all point estimates or confidence intervals were inside their limits of concern (EA_all_ method). This is a rather strict and simple assessment in which “bad” scores for an endpoint cannot be compensated by “good” scores for another endpoint. The alternative was taking the average (EA_av_); that is, bad scores for one indicator can be compensated by good scores for another. More flexible MCDA methods do exist. For example, the balance of acceptability model allows intermediate approaches between EA_all_ and EA_av_ by specification of a compensability parameter (van der Voet et al., 2014). This could perhaps be linked in future research to the ecological concept of functional redundancy, which implies that lower numbers of a particular species could be compensated, at least partly, by higher numbers of another species in the same functional group (Kang et al., 2015; Rosenfeld, 2002).

The EFSA Guidance Document (EFSA, 2010b) provides guidelines for the environmental risk assessment of GMOs. In the AMIGA project (Arpaia et al., 2014), it was found that these guidelines were frequently not specific enough, for example, regarding how to handle data from very different experiments for nonabundant species. This paper shows that a more specific protocol for the statistical analysis of such studies is feasible. Such a protocol can support a transparent analysis of nontarget organisms’ ecological data in order to evaluate equivalence. The results highlight the importance of setting limits of concern as equivalence limits for safety assessment.

## CONFLICT OF INTEREST

None declared.

## AUTHOR CONTRIBUTIONS

HvdV and PWG devised the statistical methods and calculations. HvdV wrote the draft paper. PWG performed the calculations and prepared the graphs. JL collected the arthropod count data. GJTK and EW supervised the field trials in the Netherlands and Ireland, respectively. JJAvL classified the taxa into functional groups and provided expert opinion on the limits of concern for NTO counts. SA coordinated the AMIGA project which produced this work. All authors critically revised the manuscript and approved the final version. The authors report no competing interests.

## Data Availability

Arthropod count data and analysis programs are available at the Dryad Digital Repository: https://doi.org/10.5061/dryad.72048tr.
