# Supplementary material for: Equivalence analysis to support environmental safety assessment: Using nontarget organism count data from field trials with cisgenically modified potato
Source: Ecol Evol. 2019 Feb 14;9(5):2863–82. doi: 10.1002/ece3.4964 (PMC6405891; doi:10.1002/ece3.4964)
Supplement: Supplementary file 1 [file ECE3-9-2863-s001.docx]

**Supporting Information S1**

**Statistical power of equivalence tests with adaptive limits of concern**

Supporting Information S1 to:

van der Voet H, Goedhart PW, Lazebnik J, Kessel GJT, Mullins E, van Loon JA & Arpaia, S. Equivalence analysis to support environmental safety assessment: using non-target organism count data from field trials with cisgenically modified potato.

Suppose we have two samples of size $n$ from the Poisson distribution with means $\mu_{1}$ and $\mu_{2}$, and we want to test the two-sided non-equivalence hypothesis $H_{0}: \left| \log\left( {\mu_{1}}/{\mu_{2}} \right) \right|<L$, in which $L$ is the logarithm of the limit of concern. The maximum likelihood estimator for the log-ratio $\Delta=\log\left( {\mu_{1}}/{\mu_{2}} \right)$ is given by $log({X_{m}}/{Y_{m})}$ in which $X_{m}$ and $Y_{m}$ are the respective sample means. The standard error of this estimate is given by $\sqrt{\left( 1/{X_{m}}+1/{Y_{m}} \right)/n}$. The two-sided test approach of Schuirmann (1987) for equivalence testing with significance level 0.05 employs a 90% confidence interval based on the estimate and its standard error. The null hypothesis is rejected in favour of equivalence when the confidence interval fully lies in the interval $\left( -L, L \right)$.

Now take $\mu_{1}=\mu_{2}=\mu_{0}$, where $\mu_{0}$ is the chosen limit abundance value which is used in the adaptation of the limit of concern. Further suppose that the value of $L$ is such that the power of the non-equivalence test in this case equals $\beta$. Note that $L$ is a function of $\beta$, $\mu_{0}$ and the significance level $\alpha$ which was set at 0.05, and its value is approximated by simulation. The power of the non-equivalence test employing the adaptive limit of concern $L\sqrt{{\mu_{0}}/m}$, with $m$ the mean of $X_{m}$ and $Y_{m}$, for values of $\mu_{1}=\mu_{2}=\mu$ smaller than $\mu_{0}$ can also be approximated by means of simulation.

This simulation was performed for $\mu_{0}=10$, for sample sizes $n=5, 10, 20$ for powers $\beta=50\% ,80\%$, and for $\mu=0.3, 0.4, 0.5, 0.6, 0.7, 0.8, 0.9, 1, 2, 3, 4, 5, 6, 7, 8, 9, 10$. The required value of $L$ was approximated by simulation of 500.000 datasets employing linear interpolation for values of $L$ which were 0.01 apart. Employing this value $L$, 100.000 datasets were simulated for each value of $n$ and $\mu$, and the power of the equivalence test without and with adaptive limits was approximated. The approximate values for $L$ are given in Table 1 and the simulated powers for various values of $\mu$ are given in Figure 1. This reveals that the adaptive limits of concern result in similar powers as for $\mu_{0}$. Only for small sample sizes the power is somewhat lower.

Table 1 Approximate values for $\boldsymbol{L}$ with power $\boldsymbol{\beta}$ for the non-equivalence test for $\boldsymbol{\mu}_{\boldsymbol{0}}\boldsymbol{=10}$ for the Poisson distribution

| Power $\beta$ | $n=5$ | $n=10$ | $n=20$ |
| --- | --- | --- | --- |
| 50 | 0.4670 | 0.3293 | 0.2325 |
| 80 | 0.5928 | 0.4170 | 0.2940 |


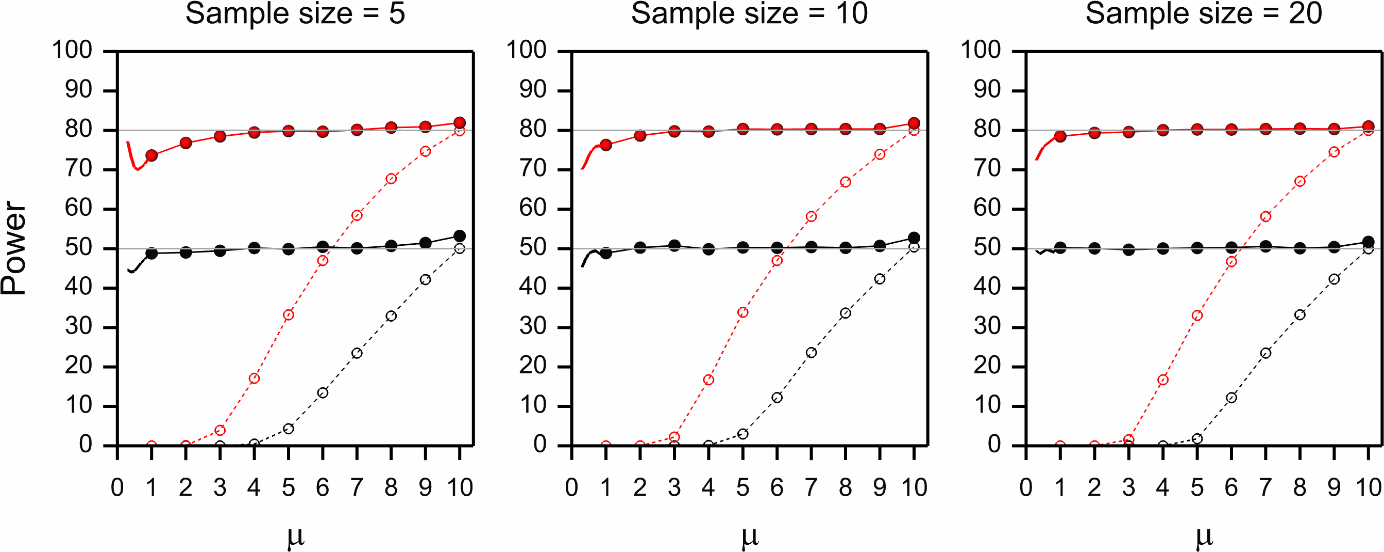


**Figure 1 Simulated powers fixed limits of concern** $\boldsymbol{L}$ **(open symbols, dotted lines) and for adaptive limits of concern (closed symbols, straight lines) for power** $\boldsymbol{\beta=50\%}$ **(black) and** $\boldsymbol{\beta=80\%}$ **(red) for the Poisson distribution.**

The same simulation was performed for the quasi-Poisson distribution with dispersion parameter $\sigma^{2}=2, 5$. The quasi-Poisson distribution arises when the Poisson distribution is mixed with the gamma distribution (Goedhart *et al.*, 2014). It is then assumed that a count *X* follows a Poisson distribution with mean *Z*, where *Z* itself follows a gamma distribution with mean $\mu$ and variance $\left( \sigma^{2}-1 \right)\mu$. The resulting distribution is a special form of the negative binomial distribution, with mean $\mu$ and variance $\sigma^{2}\mu$ (McCullagh and Nelder, 1989). Simulated data are analysed with a log-linear model employing the quasi-Poisson distribution and the dispersion parameter $\sigma^{2}$ is estimated by means of the Pearson statistic. Whenever the Pearson statistic is smaller than 1, the data are re-analysed with the Poisson distribution effectively setting the dispersion parameter $\sigma^{2}=1$. The approximate values for $L$ are given in Table 2, while the simulated powers are plotted in Figure 2. Again the simulated powers with the adaptive limits of concern are similar to those for $\mu_{0}$. The largest discrepancies are for the small sample size $n=5$ and power $\beta=50\%$.

Table 2 Approximate values for $\boldsymbol{L}$ with power $\boldsymbol{\beta}$ for the non-equivalence test for $\boldsymbol{\mu}_{\boldsymbol{0}}\boldsymbol{=10}$ for the quasi-Poisson distribution with dispersion parameter $\boldsymbol{\sigma}^{\boldsymbol{2}}$.

| Power $\beta$ | $\sigma^{2}=2$ | | | $\sigma^{2}=5$ | | |
| --- | --- | --- | --- | --- | --- | --- |
|  | $n=5$ | $n=10$ | $n=20$ | $n=5$ | $n=10$ | $n=20$ |
| 50 | 0.6595 | 0.4644 | 0.3282 | 1.0217 | 0.7282 | 0.5170 |
| 80 | 0.8558 | 0.5945 | 0.4168 | 1.3477 | 0.9394 | 0.6599 |


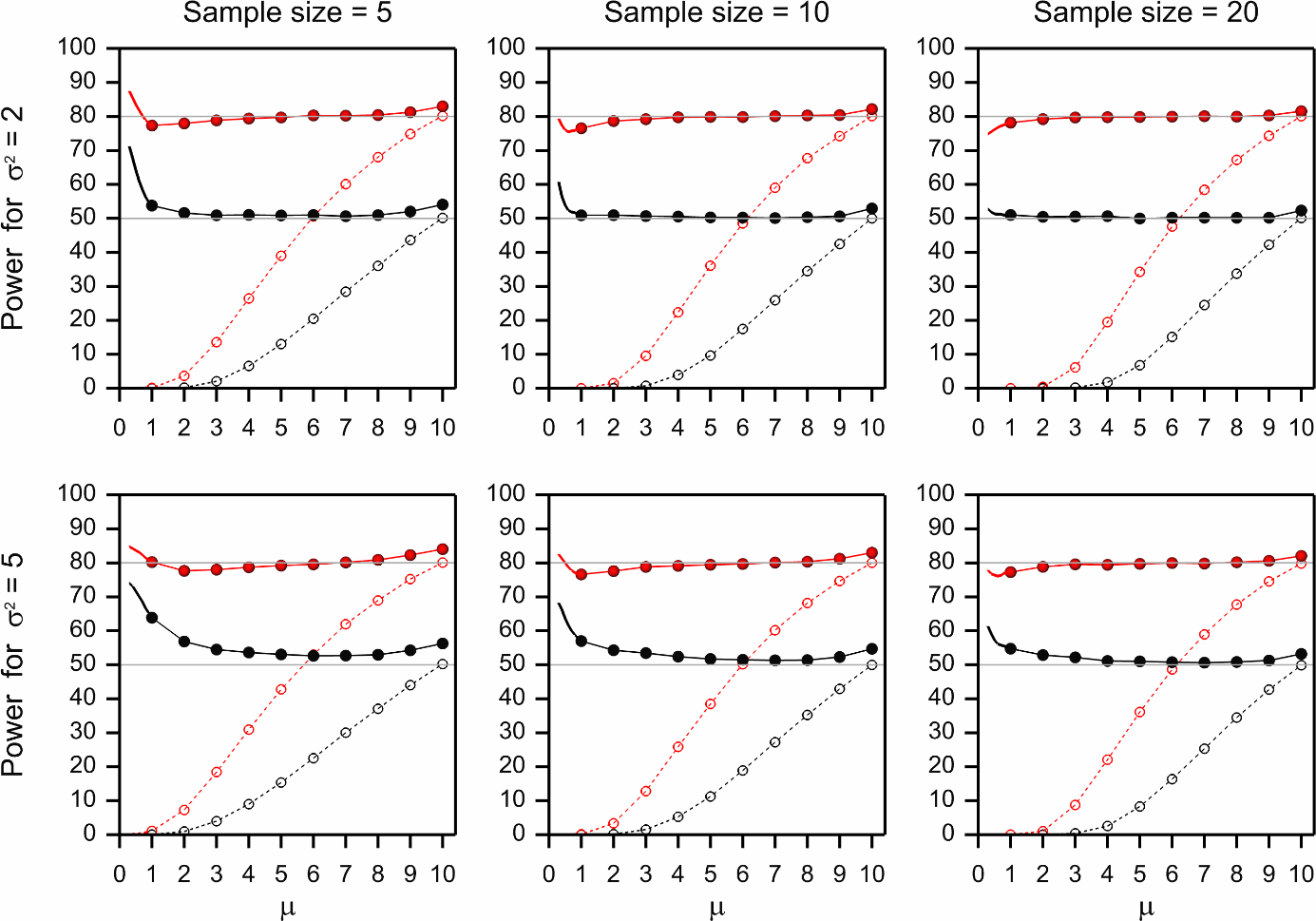


**Figure 2 Simulated powers for fixed limits of concern** $\boldsymbol{L}$ **(open symbols, dotted lines) and for adaptive limits of concern (closed symbols, straight lines) for power** $\boldsymbol{\beta=50\%}$ **(black) and** $\boldsymbol{\beta=80\%}$ **(red) for the quasi-Poisson distribution with** $\boldsymbol{\sigma}^{\boldsymbol{2}}\boldsymbol{=2}$ **(top panels) and** $\boldsymbol{\sigma}^{\boldsymbol{2}}\boldsymbol{=5}$ **(bottom panels).**

**References**

Goedhart PW, van der Voet H, Baldacchino F & Arpaia S (2014). A statistical simulation model for field testing of non-target organism in environmental risk assessment of genetically modified plants. *Ecology and Evolution*, 4(8), 1267-1283. <https://doi.org/10.1002/ece3.1019>

McCullagh P & Nelder JA (1989). *Generalized Linear Models*. Chapman and Hall. London.

Schuirmann DJ (1987). A comparison of the two one‑sided tests procedure and the power approach for assessing the equivalence of average bioavailability. *Journal of Pharmacokinetics and Biopharmaceutics*, 15(6): 657‑680. <https://doi.org/10.1007/BF01068419>
