# Supplementary material for: Equivalence analysis to support environmental safety assessment: Using nontarget organism count data from field trials with cisgenically modified potato
Source: Ecol Evol. 2019 Feb 14;9(5):2863–82. doi: 10.1002/ece3.4964 (PMC6405891; doi:10.1002/ece3.4964)
Supplement: Supplementary file 2 [file ECE3-9-2863-s002.docx]

**Supporting Information S2**

**Differences per taxon on the LoCSDIF and CQ scales**

Supporting Information S2 to:

van der Voet H, Goedhart PW, Lazebnik J, Kessel GJT, Mullins E, van Loon JA & Arpaia, S. Equivalence analysis to support environmental safety assessment: using non-target organism count data from field trials with cisgenically modified potato.


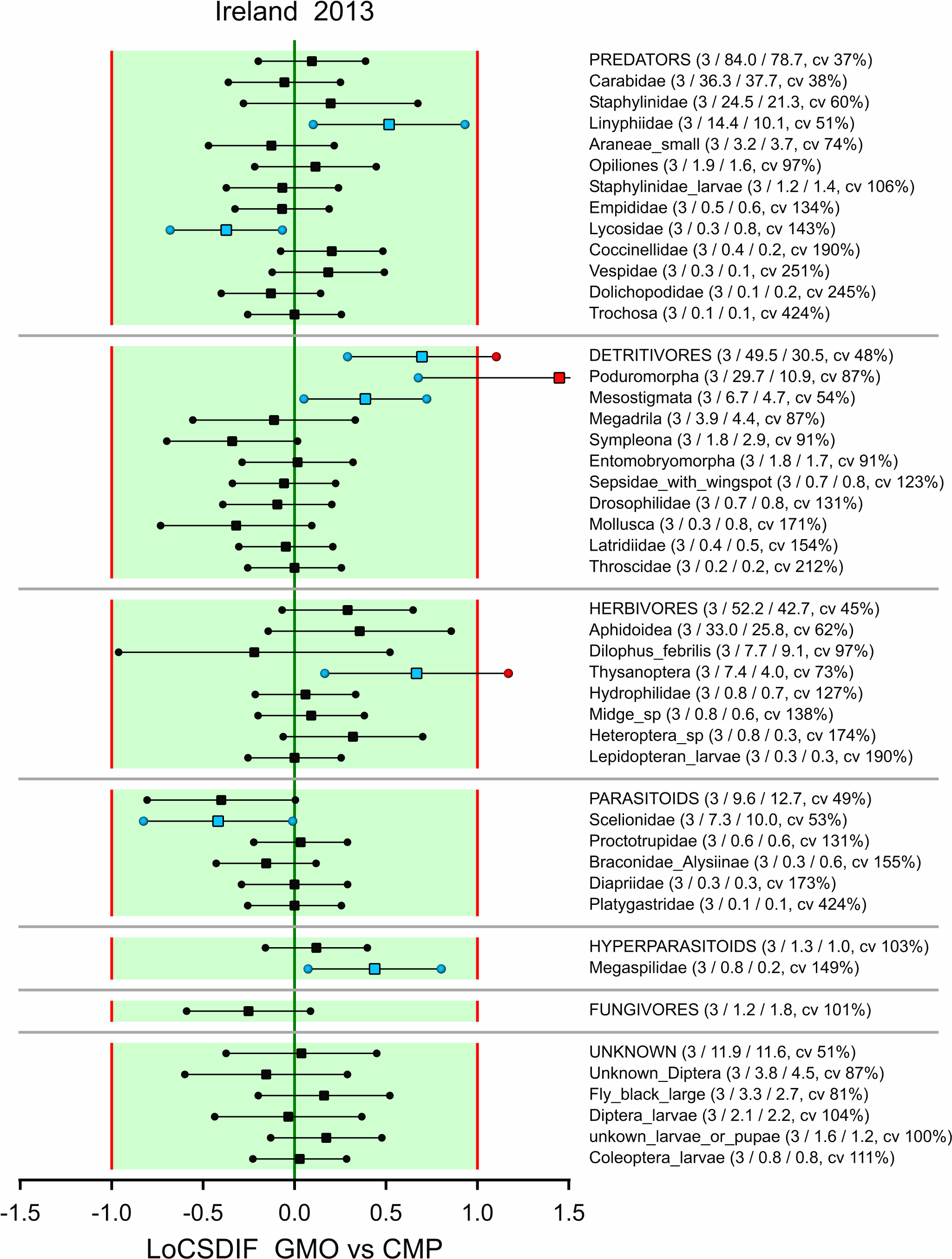


Figure S2-1. Arthropods in potato trial in Ireland 2013. LoCSDIF 90% confidence intervals for GMO vs CMP averaged over Control strategies if possible. Added in parentheses are the number of Control strategies over which is averaged, the means for the GMO and CMP and the coefficient of variation (cv).


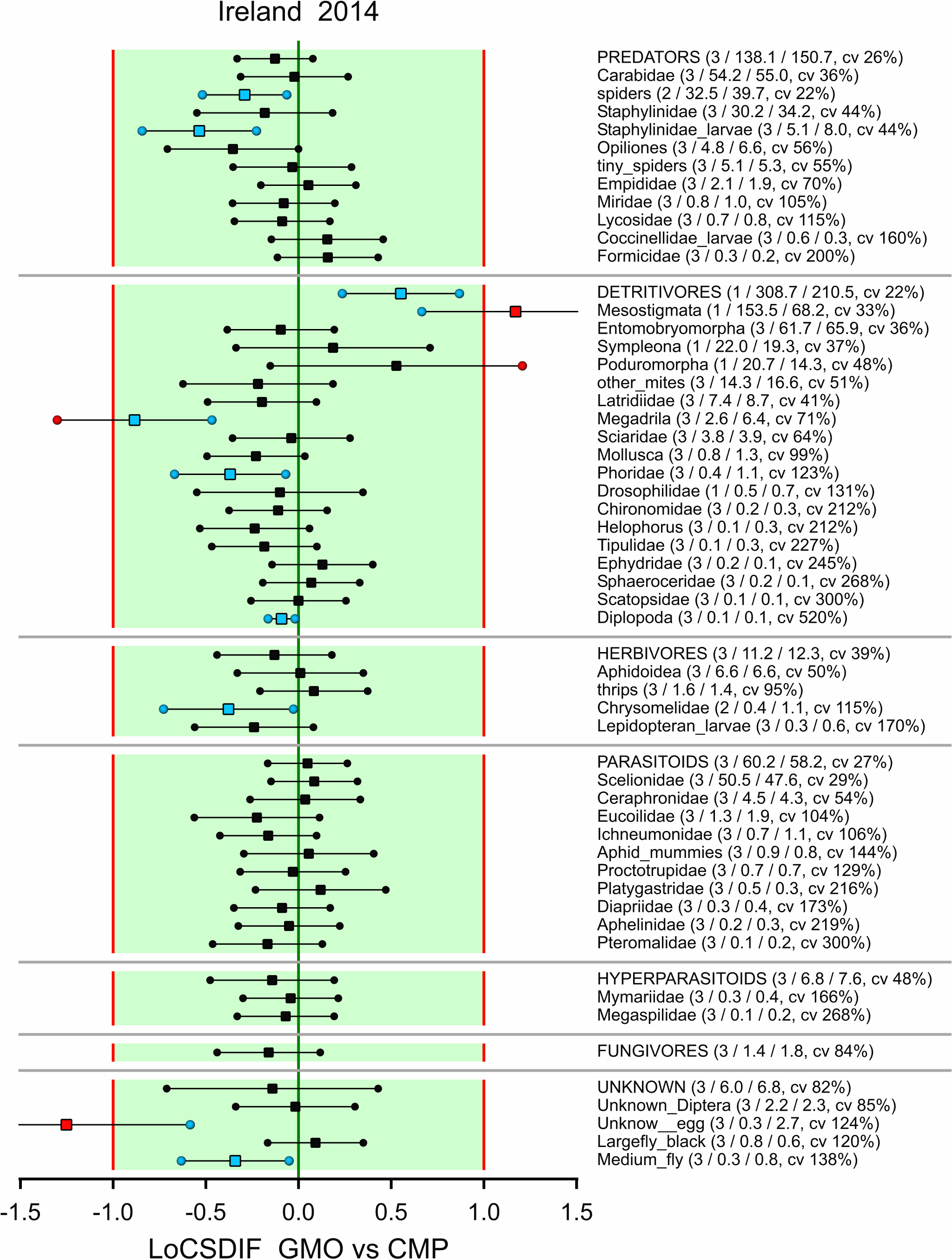


Figure S2-2. Arthropods in potato trial in Ireland 2014. LoC-scaled difference 90% confidence intervals for GMO vs CMP averaged over Control strategies if possible. Added in parentheses are the number of Control strategies over which is averaged, the means for the GMO and CMP and the coefficient of variation (cv).


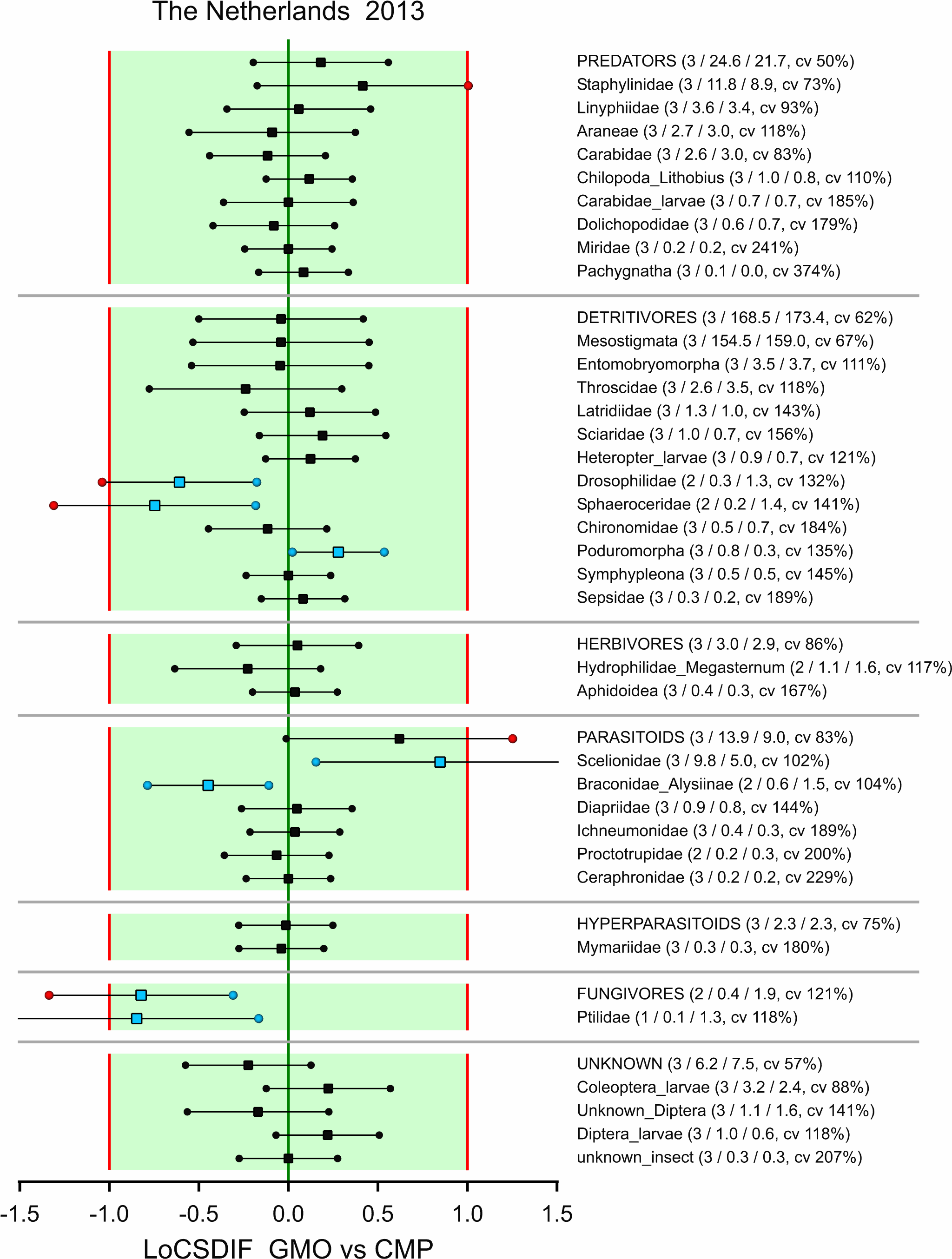


Figure S2-3. Arthropods in potato trial in The Netherlands 2013. LoC-scaled difference 90% confidence intervals for GMO vs CMP averaged over Control strategies if possible. Added in parentheses are the number of Control strategies over which is averaged, the means for the GMO and CMP and the coefficient of variation (cv).


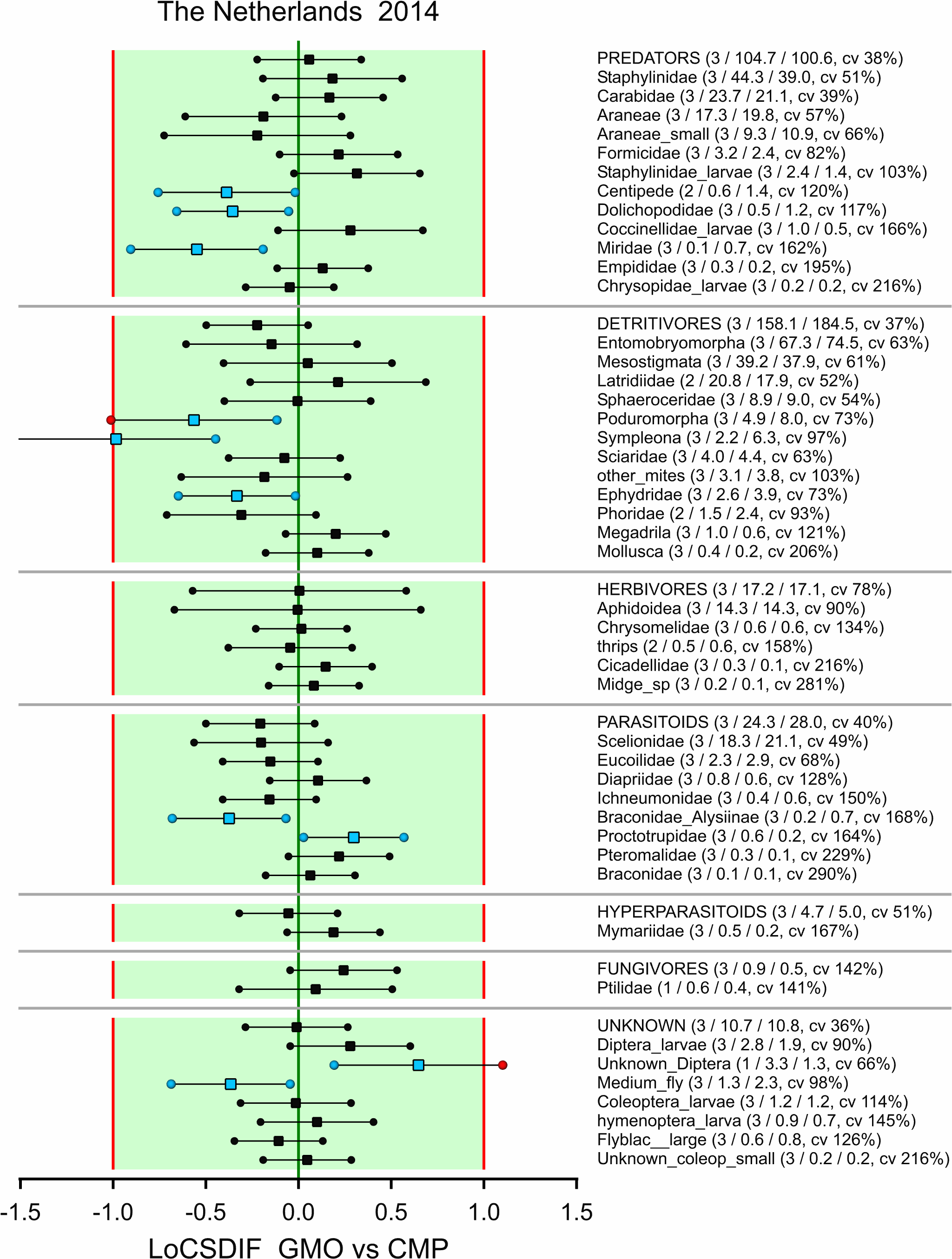


Figure S2-4. Arthropods in potato trial in The Netherlands 2014. LoC-scaled difference 90% confidence intervals for GMO vs CMP averaged over Control strategies if possible. Added in parentheses are the number of Control strategies over which is averaged, the means for the GMO and CMP and the coefficient of variation (cv).


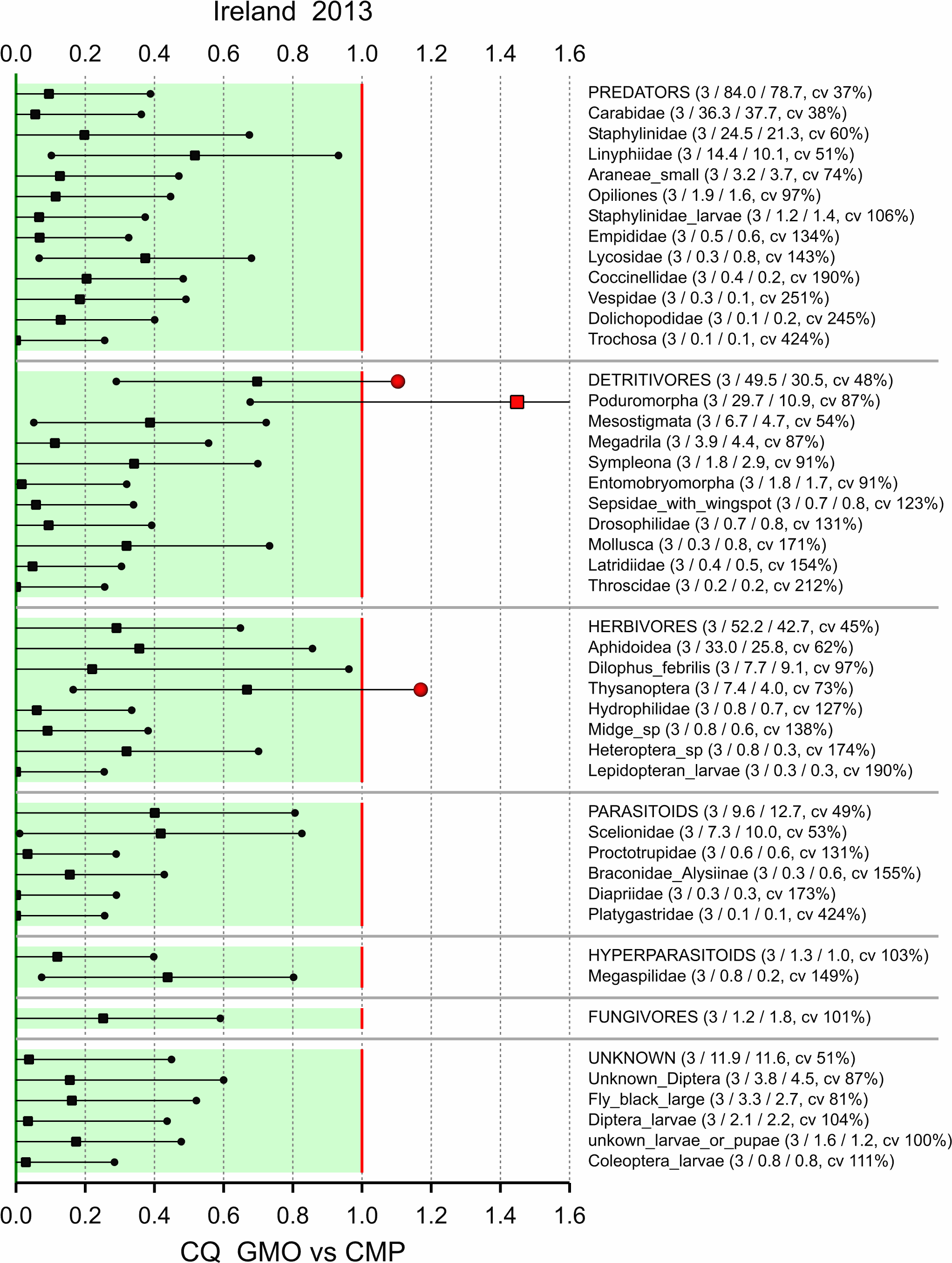


Figure S2-5. Arthropods in potato trial in Ireland 2013. Concern Quotient 90% confidence intervals for GMO vs CMP averaged over Control strategies if possible. Added in parentheses are the number of Control strategies over which is averaged, the means for the GMO and CMP and the coefficient of variation (cv).


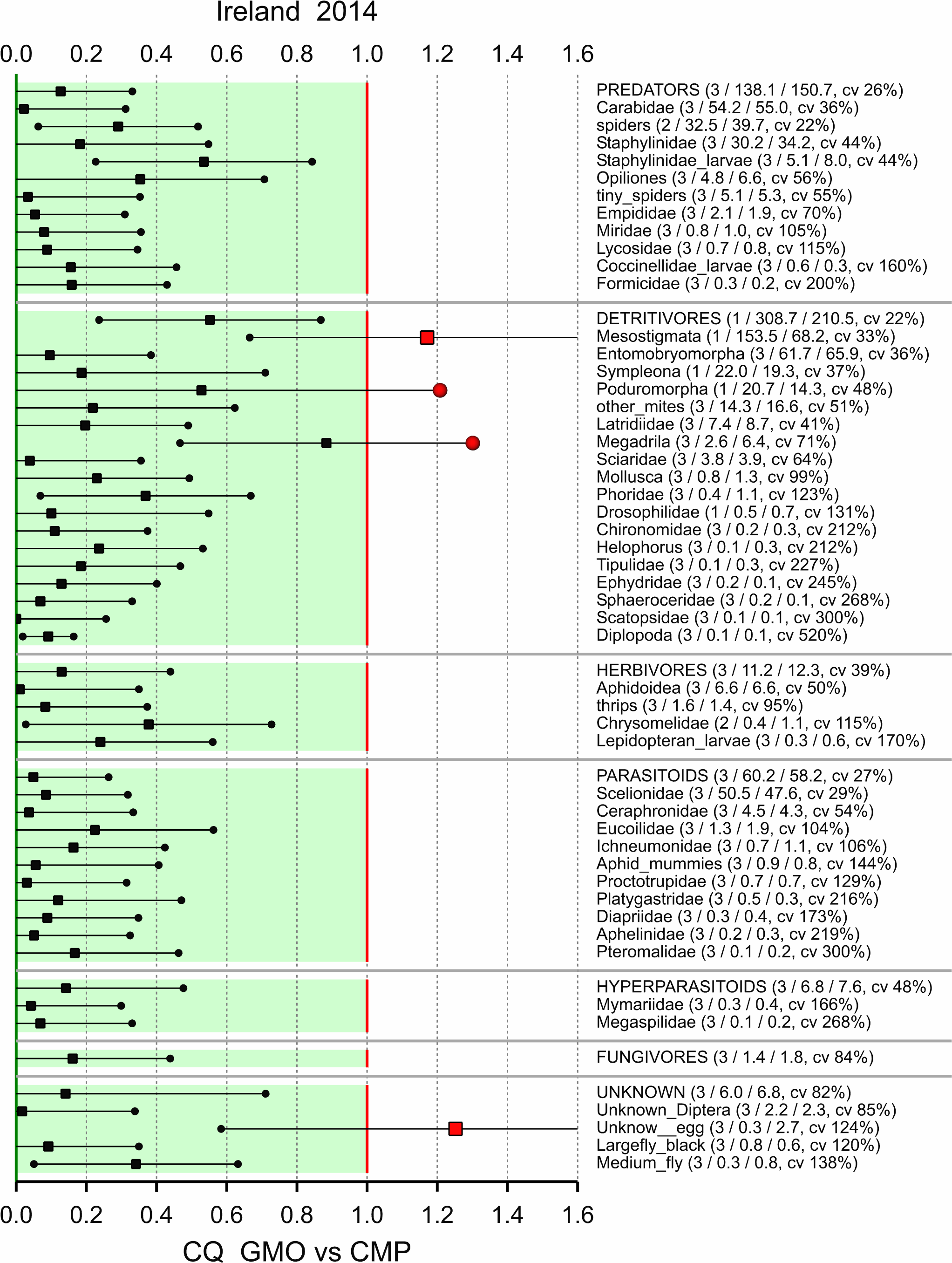


Figure S2-6. Arthropods in potato trial in Ireland 2014. Concern Quotient 90% confidence intervals for GMO vs CMP averaged over Control strategies if possible. Added in parentheses are the number of Control strategies over which is averaged, the means for the GMO and CMP and the coefficient of variation (cv).


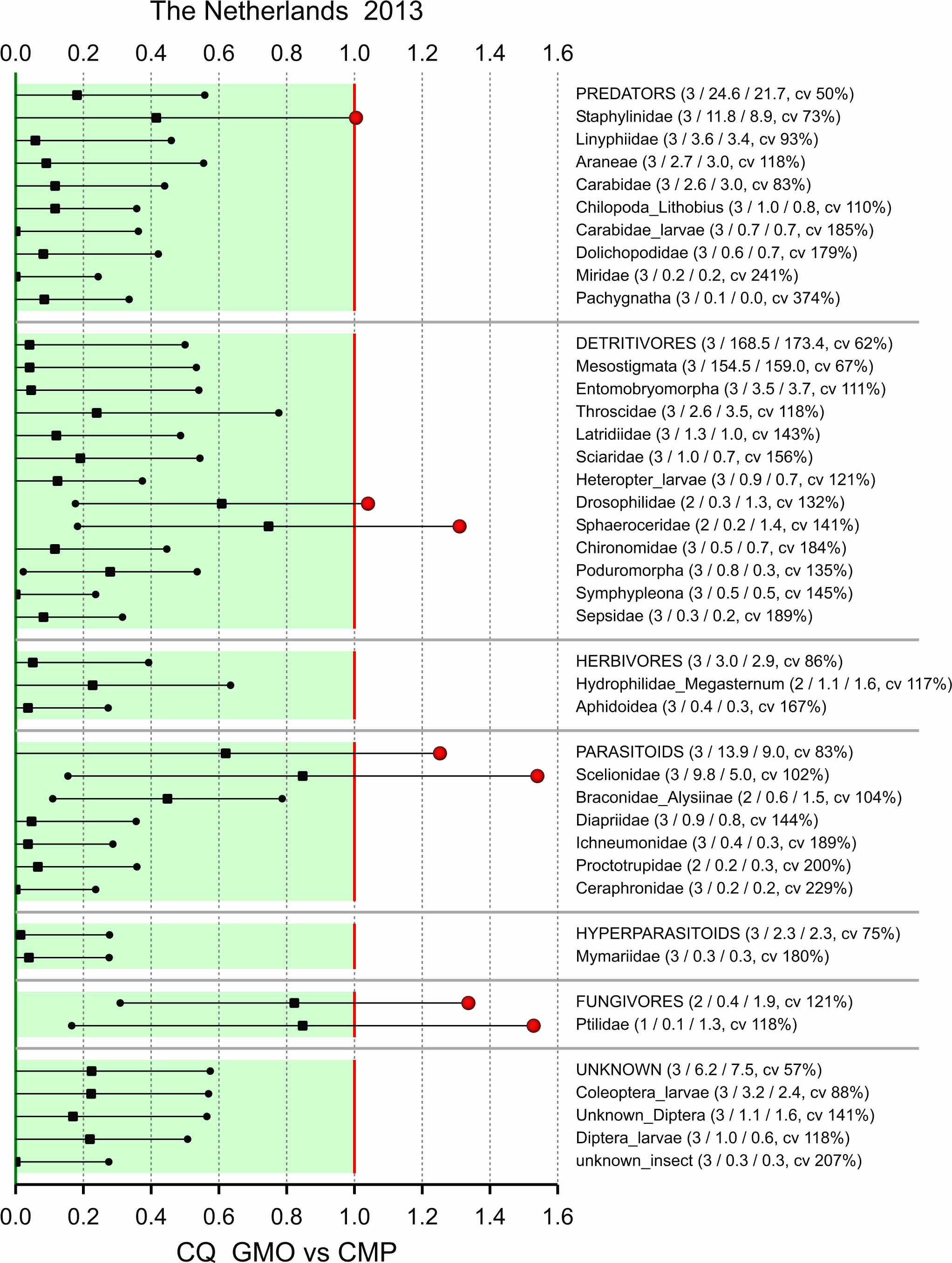


Figure S2-7. Arthropods in potato trial in The Netherlands. Concern Quotient 90% confidence intervals for GMO vs CMP averaged over Control strategies if possible. Added in parentheses are the number of Control strategies over which is averaged, the means for the GMO and CMP and the coefficient of variation (cv).


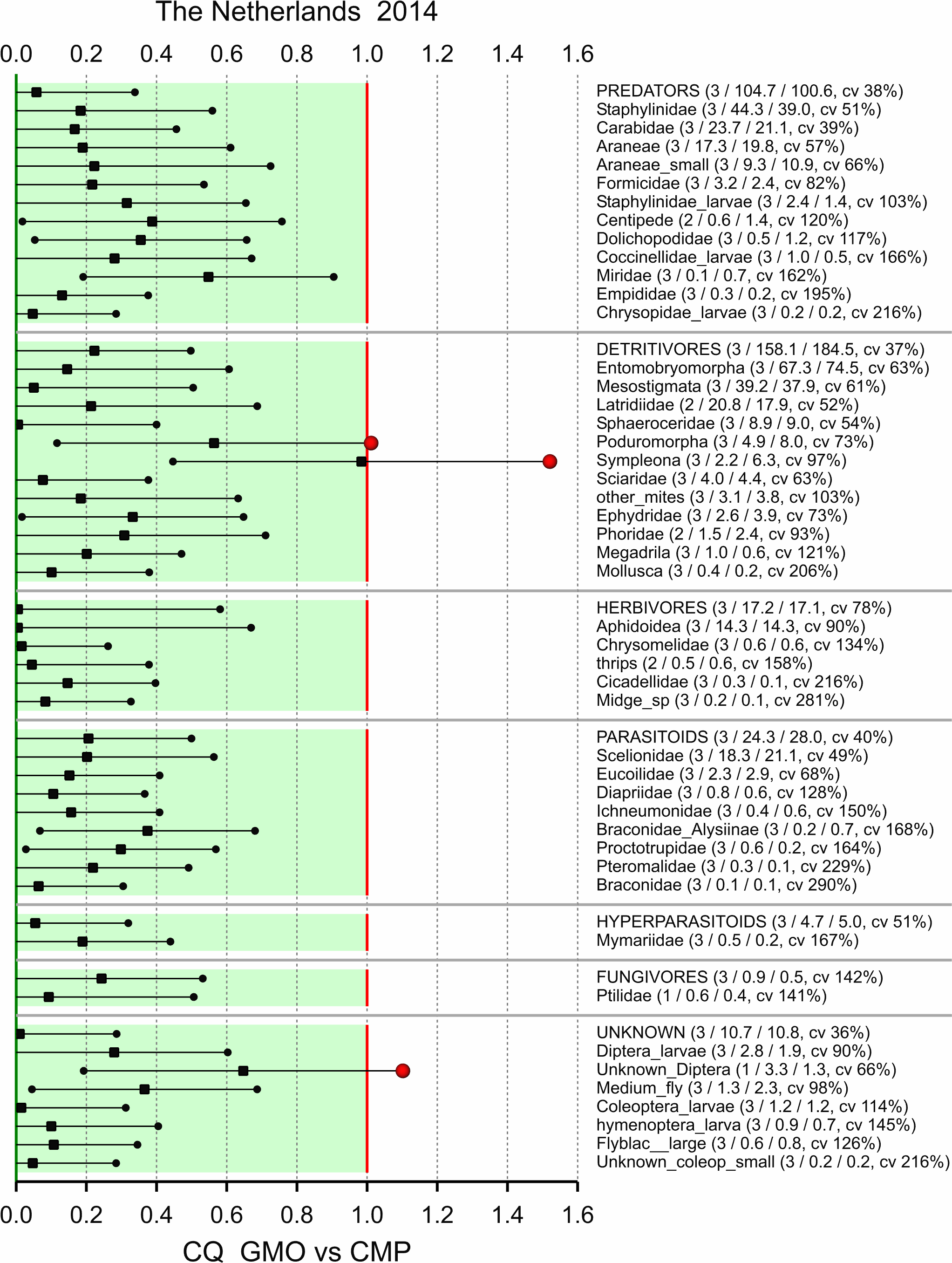


Figure S2-8. Arthropods in potato trial in The Netherlands. Concern Quotient 90% confidence intervals for GMO vs CMP averaged over Control strategies if possible. Added in parentheses are the number of Control strategies over which is averaged, the means for the GMO and CMP and the coefficient of variation (cv).
